# Design and Evaluation of Synthetic RNA-Based Incoherent Feed-Forward Loop Circuits

**DOI:** 10.3390/biom11081182

**Published:** 2021-08-10

**Authors:** Seongho Hong, Dohyun Jeong, Jordan Ryan, Mathias Foo, Xun Tang, Jongmin Kim

**Affiliations:** 1 Department of Life Sciences, Pohang University of Science and Technology, Pohang 37673, Korea; shhong1205@postech.ac.kr (S.H.); gyu9506@postech.ac.kr (D.J.); 2 Cain Department of Chemical Engineering, Louisiana State University, Baton Rouge, LA 70803, USA; jryan34@lsu.edu; 3 School of Engineering, University of Warwick, Coventry CV4 7AL, UK

**Keywords:** RNA-based regulators, small transcriptional activating RNA (STAR), toehold switch (THS), three-way junction (3WJ) repressor, type 1 IFFL, pulse generation

## Abstract

RNA-based regulators are promising tools for building synthetic biological systems that provide a powerful platform for achieving a complex regulation of transcription and translation. Recently, de novo-designed synthetic RNA regulators, such as the small transcriptional activating RNA (STAR), toehold switch (THS), and three-way junction (3WJ) repressor, have been utilized to construct RNA-based synthetic gene circuits in living cells. In this work, we utilized these regulators to construct type 1 incoherent feed-forward loop (IFFL) circuits in vivo and explored their dynamic behaviors. A combination of a STAR and 3WJ repressor was used to construct an RNA-only IFFL circuit. However, due to the fast kinetics of RNA–RNA interactions, there was no significant timescale difference between the direct activation and the indirect inhibition, that no pulse was observed in the experiments. These findings were confirmed with mechanistic modeling and simulation results for a wider range of conditions. To increase delay in the inhibition pathway, we introduced a protein synthesis process to the circuit and designed an RNA–protein hybrid IFFL circuit using THS and TetR protein. Simulation results indicated that pulse generation could be achieved with this RNA–protein hybrid model, and this was further verified with experimental realization in *E. coli*. Our findings demonstrate that while RNA-based regulators excel in speed as compared to protein-based regulators, the fast reaction kinetics of RNA-based regulators could also undermine the functionality of a circuit (e.g., lack of significant timescale difference). The agreement between experiments and simulations suggests that the mechanistic modeling can help debug issues and validate the hypothesis in designing a new circuit. Moreover, the applicability of the kinetic parameters extracted from the RNA-only circuit to the RNA–protein hybrid circuit also indicates the modularity of RNA-based regulators when used in a different context. We anticipate the findings of this work to guide the future design of gene circuits that rely heavily on the dynamics of RNA-based regulators, in terms of both modeling and experimental realization.

## 1. Introduction

Synthetic biology is a nascent discipline that integrates various research fields, including biotechnology, molecular biology, and biophysics, which aims to build artificial decision-making circuits in living organisms in a predictable and programmable manner [1,2]. Over the past decades, synthetic circuits such as oscillators [3,4], bistable switches [5,6] and arithmetic circuits [7,8] have been constructed in bacteria and mammalian cells to demonstrate the programmability of synthetic gene circuits. These developments pave the way to novel applications of synthetic gene circuits in fields that require a precise control of gene expression, such as artificial chemical production in biofoundry [9], immunomodulation-based cell therapy [10] and biosensor development [11].

**Figure 1:**
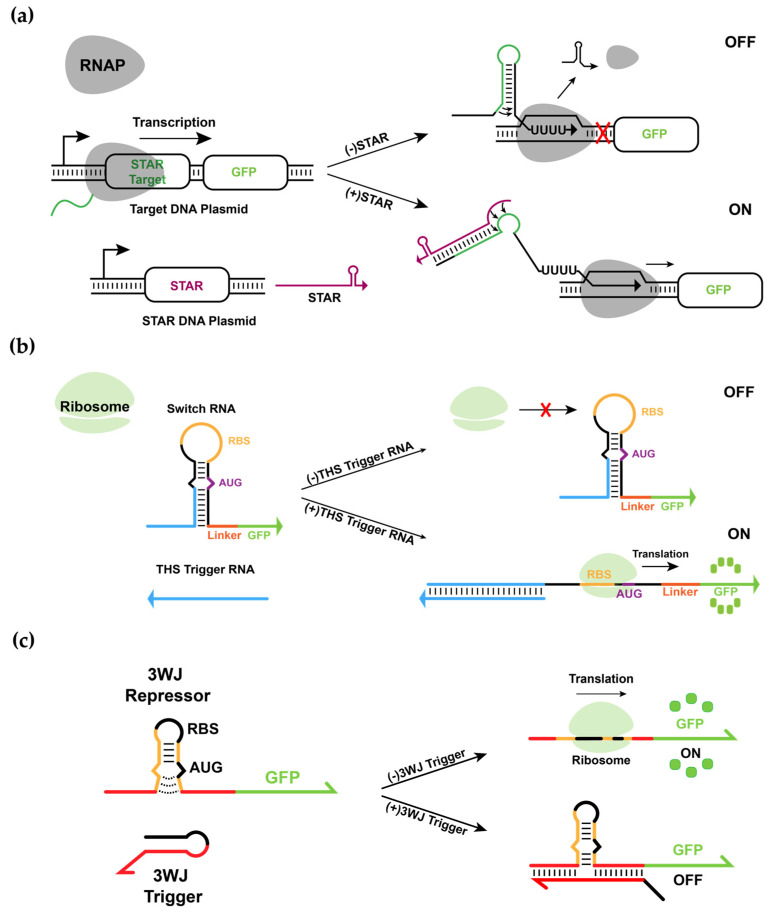
De novo-designed synthetic RNA regulators, STAR, THS and 3WJ repressor. (**a**) Schematic of the STAR mechanism. The target RNA folds into a rho-independent transcription terminator that causes RNA polymerase (RNAP) to halt transcription. When STAR binds to the target RNA, terminator formation is prevented, allowing transcription elongation of the gene. (**b**) Schematic of the THS mechanism. The ribosomal binding site (RBS) and start codon (AUG) of the switch RNA are exposed only when the cognate trigger RNA disrupts the secondary structure. (**c**) The switch RNA of a 3WJ repressor contains an unstable hairpin structure that allows ribosomal access to the RBS and start codon and toehold domains for 3WJ trigger binding. The 3WJ trigger RNA employs a toehold to bind to the 3WJ repressor RNA, forming a 3WJ structure that prevents translation initiation.

Nucleic acid-based regulators are promising tools for building synthetic biological systems due to their excellent programmability and predictable thermodynamic properties [12]. In particular, RNA-based regulators provide advantages over protein-based regulators such as a faster signal propagation through rapid degradation rates and less burden on cellular resources [13,14]. RNA regulatory components provide a robust platform for achieving a complex regulation of transcription and translation, as well as mRNA degradation [15]. Recently, de novo-designed synthetic RNA regulators, such as small transcriptional activating RNA (STAR) [16] and toehold switch (THS) [17] have been utilized at the transcription and the translation level. These RNA regulators offer a promising toolbox with a large library size and wide dynamic ranges. In the STAR mechanism, a target RNA, derived from the intrinsic transcriptional terminator, prevents the transcription of the downstream gene when the terminator structure is formed (Figure 1a). When trans-acting antisense RNA complementarily binds to the 5′ region of the target RNA, it inhibits terminator formation, allowing the transcription of downstream genes. On the other hand, THS is a riboregulator that regulates translation initiation by sequestering the start codon and ribosome binding site (RBS) into a stable RNA secondary structure (Figure 1b). Translation of the downstream gene can be initiated only if the switch RNA stem is unwound by the cognate trigger RNA. THS exhibits high ON/OFF ratios and has been extended to multi-input logic-processing ribocomputing devices such as AND/OR/NOT logic gates [18]. Recent work has further expanded the capability of THS to NAND and NOR logic gates with RNA-based repressors called three-way junction (3WJ) repressors [19]. The 3WJ repressor can be easily translated due to the unstable hairpin structure around the RBS and start codons, but the translation is inhibited in the presence of a cognate trigger through the formation of a stable three-way junction structure (Figure 1c). Together, these de novo-designed RNA elements provide modular parts to construct RNA-based synthetic gene circuits in living cells.

The type 1 incoherent feed-forward loop (IFFL) network motif is commonly found in natural bacterial networks and has received interest due to its wide applications such as band-pass filters, fold-change detection [20,21], biosensing, and engineered promoter maintaining a constant level of gene expression at any copy number [22]. The IFFL may be prevalent because of its small size and stability: feedback loops with delays often result in an unstable outcome, while the IFFL with delays produces pulsed gene expression, which can be linked to diverse processes in living organisms ranging from stress response, signaling, and development [23,24]. An IFFL is composed of two incoherent regulation pathways on the target gene Z, with a direct activation from X and an indirect repression from X via an X-activated intermediate repressor Y. When Z is used as a reporter for the circuit output, it is possible to generate a pulse of gene Z expression because the activation of Z is initiated immediately by X, whereas the inhibition of Z occurs with a delay due to the presence of the intermediate component Y. Recent works demonstrated several ways to implement IFFL circuits with DNA strand displacement mechanisms [21], CRISPR interference (CRISPRi) [25,26], and microRNAs [27]. However, the systematic construction and detailed analysis of RNA-based synthetic IFFL circuits with the ability to show pulsed gene expression in vivo has not been clearly demonstrated.

Here, we employ STARs to realize the direct activation of gene expression at the transcription level, and 3WJ repressors to achieve repression at the translation level for an RNA-only IFFL circuit. In a similar vein, we also explore an RNA–protein hybrid IFFL circuit with THS and TetR protein [28]. Gene expression of TetR (Y) and GFPmut3b-ASV (Z) can both be activated by the regulator toehold trigger (X), and TetR (Y) can then bind to the operator site in the promoter region of Z to repress the transcription of Z. These different IFFL circuits provide interesting case studies to evaluate the application of different RNA-based regulators in the design of dynamical circuits in vivo. The simple, yet interesting, dynamics of the IFFLs are also amenable for mathematical modeling and analysis.

Mathematical modeling validated with experimental measurements can provide a rapid characterization of parts and a reliable prediction of circuit performance under a broad range of conditions that are challenging and costly to achieve by experiments. The success of using mathematical modeling to quantify and guide the design of synthetic gene circuits has been reported in various systems from bacteria to mammalian cells [29,30,31,32,33]. Given previous successes with model-guided design with the evaluation of the gene pulse generator and IFFL circuits [25,34,35,36], here, we develop ordinary differential equation (ODE)-based mathematical models to first explore the capability of an RNA-only circuit functioning as an IFFL circuit for pulse generation and then, to guide the design and experimental realization of an RNA–protein hybrid IFFL circuit with predictable dynamics. The ODE models in this study feature both the mechanistic description of the chemical reactions in the system based on first principles and the phenomenological description of the dynamics with Hill-type functions.

Here, we started by constructing an RNA-only IFFL circuit and analyzing its dynamics in *E. coli*. Then, we constructed mathematical models to perform a more comprehensive investigation of the dynamics of the RNA-only IFFL circuit, under different conditions. The experiment and simulation indicate that the RNA-only IFFL circuit may not have enough delays between the activation and the inhibition pathways for a pronounced pulse generation and confirm the importance of a timescale difference in IFFL, as previously reported [25,37]. Based on this observation, we then proposed and developed a mathematical model for an RNA–protein hybrid IFFL circuit which showed pulse generation within plausible parameter ranges. The corresponding molecular circuit tested in *E. coli* also showed pulse generation for a wide range of inducer concentrations, demonstrating a good agreement with the mathematical model. Our results demonstrate that the combination of modeling and experiments is an effective approach to prototyping synthetic RNA circuits for the predictable and precise control of gene expression. The findings in our study also provide evidence that RNA-based circuits can serve as modular and composable parts for synthetic biological circuits.

## 2. Materials and Methods

### 2.1. Plasmid Construction and E. coli Strains Used

Backbones for the plasmids were taken from the commercial vectors pET15b, pCDFDuet, and pCOLADuet (EMD Millipore, Billerica, MA, USA). Node X was constructed in pET15b. Node Y and node Z were constructed in pCDFDuet and pCOLADuet. All constructs were cloned via Gibson assembly and round-the-horn site-directed mutagenesis, and detailed protocol can be found in Supplementary Information. Plasmid architecture and specific part sequences are listed in Tables S4–S9. Plasmids were purified using Enzynomics EZ-Pure Plasmid Prep Kit (Catalog number: EP101-200N). Plasmid sequences were confirmed by DNA sequencing. Plasmids were transformed into strains via chemical transformation. *E. coli* BL21 AI strain for RNA-only IFFL circuit and *E. coli* BL21 DE3 strain for RNA–protein hybrid IFFL circuit were used for in vivo tests.

### 2.2. Cell Culture and Microplate Reader Analysis

Transformed cells were cultured on Luria-Bertani (LB) agar plates (1.5% agar) and then single colonies were inoculated into 500 µL LB liquid medium supplemented with appropriate antibiotics: pCOLADuet (50 μg/mL Kanamycin), pCDFDuet (50 μg/mL Spectinomycin), pET15b (100 μg/mL Ampicillin). These cells were grown overnight (~16 h) in 96-well plates with shaking at 800 r.p.m. and 37 °C. For the RNA-only IFFL circuit, overnight cultures were diluted 1/100-fold, and then Isopropyl β-d-1-thiogalactopyranoside (IPTG) and Arabinose were treated immediately. IPTG was treated at four different concentrations: 500 µM, 125 µM, 31.2 µM, 7.81 µM. Arabinose was treated at four different concentrations: 6600 μM, 1650 μM, 412.5 μM, 103.1 μM. For the RNA–protein hybrid IFFL circuit, overnight cultures were diluted 1/50-fold, and then IPTG and anhydrotetracycline (aTc) were treated immediately. IPTG was treated at four different concentrations: 1 mM, 0.2 mM, 0.1 mM, 0.02 mM. aTc was treated at six different concentrations: 400 nM, 200 nM, 40 nM, 4 nM, 2 nM (200 ng/mL, 100 ng/mL, 20 ng/mL, 10 ng/mL, 2 ng/mL, 1 ng/mL). An aliquot of 200 µL of inducer-treated cells was added per well on a 96-well Black Plate (SPL Life Sciences, Pocheon, Korea). Plates were incubated at 37 °C with double-orbital shaking in a Synergy H1 microplate reader (BioTek, Winooski, VT, USA) running Gen5 3.08 software. GFP Fluorescence (excitation: 479 nm, emission: 520 nm) and OD600 were measured at 10-min intervals during incubation. The RNA-only IFFL circuit was incubated for 5 h (31 cycles), and the RNA–protein hybrid-based IFFL circuit was incubated for 8 h (49 cycles). GFP fluorescence levels were normalized as follows: GFP fluorescence for LB blank was subtracted and the resulting value was divided by OD600. The number of replicates was three for RNA-only and six for RNA–protein hybrid IFFL circuits.

### 2.3. Mathematical Modeling

The mathematical ODE models were solved with MATLAB *odes23s* solver for the simulated GFP concentration, in both the model parameterization with experimental data, and the sensitivity analysis for the RNA–protein hybrid model. Parameterization of the RNA-only and RNA–protein hybrid models were performed by fitting to the experimental measurements with MATLAB functions *fminsearch* and *fmincon*, respectively. The sensitivity analysis was completed using MATLAB random number generation function *rand* to randomly select kinetic parameters within the perturbed range, as described in detail in the modeling section below. All the related MATLAB scripts for this work are available at https://github.com/mathiasfoo/ifflbiomolecule (accessed on 22 July 2021).

## 3. Results

### 3.1. Design and Experimental Characterization of Synthetic RNA-Based IFFL Circuit

We first sought to construct the RNA-only IFFL circuit in *E. coli*. To this end, we designed a three-component circuit where the top regulator, X, generated a trans-acting RNA that controlled STAR elements in the two downstream nodes, Y and Z. The intermediate node Y, in turn, expresses an inhibitor for Z, which contains a 3WJ repressor switch module that can be inhibited by a 3WJ trigger RNA. The 3WJ trigger from Y binds to the bottom of the weak stem domain in the 3WJ repressor switch in Z to terminate the translation of GFP reporter protein. Thus, in the system we designed, X activated Z directly and inhibited Z indirectly through an intermediate node Y to complete the IFFL circuit.

**Figure 2:**
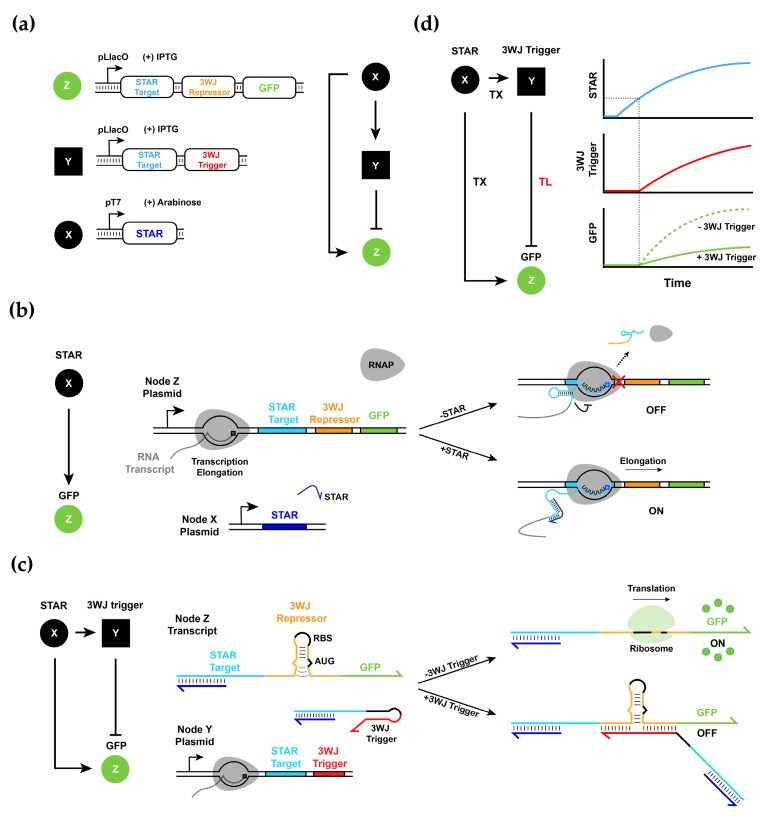
Architecture of RNA-only IFFL circuit composed of STAR and 3WJ repressor. (**a**) Detailed node constructs of RNA-only IFFL circuit. Node Z contains the STAR target linked to 3WJ repressor and GFP under the control of pLlacO. Node Y contains the STAR target linked to 3WJ trigger under the control of pLlacO. Node X contains the STAR trigger expressed under pT7. (**b**) Schematic of the activation by X on Z. When X binds to the STAR target of Z, terminator formation is prevented, allowing transcription elongation. (**c**) Schematic of the inhibition by Y on Z. Y, which is transcribed in the presence of X, can bind to the 3WJ repressor switch domain of Z, forming a 3WJ structure that prevents translation initiation. (**d**) Expected GFP expression profile in the presence and absence of the 3WJ trigger activated by STAR. The 3WJ trigger may be able to repress GFP expression by forming 3WJ structures with little time delay. Note, TX stands for transcription and TL stands for translation.

At the molecular level, we used a three-plasmid system where X, Y, and Z each corresponded to a different plasmid to allow different combinations of circuit components to be easily tested in *E. coli*. X contained a trans-acting RNA under the control of the T7 promoter (pT7), Y contained a STAR connected to a 3WJ trigger under the control of the Lac promoter (pLlacO), and Z contained a STAR connected to a 3WJ switch and a GFP reporter under the control of pLlacO (Figure 2a). The use of different promoters allowed us to control the number of transcripts of X, Y, and Z with different inducers: Arabinose and IPTG. In our system, since the T7 RNAP expression was controlled by Arabinose in the BL21 AI strain, different Arabinose concentrations could then be used to control the amount of trans-acting RNA from X. Similarly, the Lac repressor control could be relieved by IPTG such that the promoter activity of Y and Z was controlled together.

To test the performance of STAR and trans-acting RNA, we first conducted an activation experiment with input node X and output node Z. Upon the production of trans-acting RNA from X, the transcription of Z was initiated (Figure 2b). Since Z contained the STAR target linked to the 3WJ repressor-GFP as a reporter module, and the 3WJ trigger was not present in the system, the GFP fluorescence increased with an increasing concentration of both inducers Arabinose and IPTG, as expected (Figure S1). Note that at very high inducer levels, the response of the molecular circuit saturated and no further increase in GFP fluorescence outputs was observed.

Next, we included all three plasmids in *E. coli* and tested the response of the circuit for different inducer concentrations. Since Y contained a STAR target connected to the 3WJ trigger, the production of the 3WJ trigger occurred right upon activation via trans-acting RNA for STAR from X. In that case, while transcription from Z was activated by trans-acting RNA, the translation of the GFP reporter could be inhibited by the 3WJ trigger from Y (Figure 2c). We expected that as X reached a certain concentration level, transcription from nodes Y and Z was activated. Upon the build-up of 3WJ repressors from node Y, the translation of the GFP reporter from Z was inhibited (Figure 2d). We expected that the speed of the direct activation by X on Z was faster than the speed of the indirect inhibition through Y and, thus, the circuit had the potential to show a pulse in the GFP fluorescence signal.

To prove this hypothesis in vivo, the X, Y, and Z plasmids were simultaneously transformed into the *E. coli* BL21 AI strain. While the strength of expression levels from X could be controlled by Arabinose, the expression levels of Y and Z were simultaneously controlled by IPTG. Four different variants of Y were designed to control the connection strength of the indirect regulatory path: regular Y (the 3WJ trigger immediately after the STAR element under pLlacO), insulated Y (the 3WJ trigger connected via ribozyme [38] after the STAR element under pLlacO), Decoy (an unrelated control sequence under pLlacO), and constitutive (a 3WJ trigger under pLlacO) (Figure S2). The GFP outputs from the circuits were expected to decrease as the strength of inhibition increased. A strong GFP expression was observed in the absence of a 3WJ trigger (Decoy) and a very low GFP signal is observed for a constitutive 3WJ trigger (Figure S3). An intermediate signal level was achieved for regular Y, which contains 3WJ trigger, whereas the inhibition by insulated Y decreased GFP signal to very low levels close to that achieved by a constitutive 3WJ trigger expression possibly due to release of 3WJ triggers via ribozyme-mediated cleavage.

**Figure 3:**
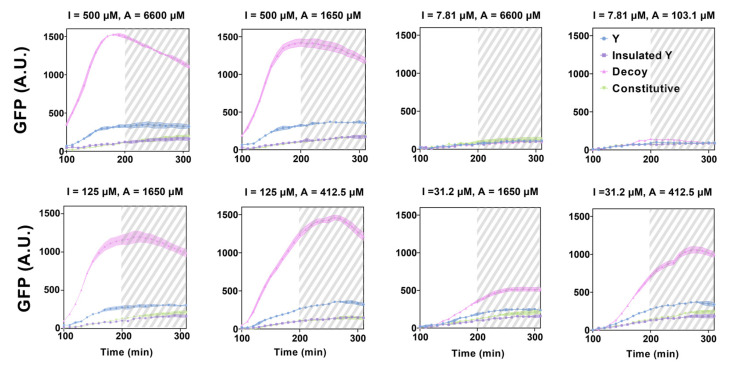
In vivo characterization of the RNA-only IFFL circuit composed of STAR and 3WJ repressor. Time course of GFP fluorescence measurements for different inducer concentrations; IPTG concentrations at 500 μM, 125 μM, 31.2 μM and 7.81 μM; Arabinose concentrations at 6600 μM, 1650 μM, 412.5 μM and 103.125 μM. Data for the first 90 min are removed due to low OD600 values, and the time points beyond 200 min are marked as gray dashed area to indicate the transition to stationary phase. Regular Y is represented by blue circles, insulated Y by purple squares, Decoy by pink triangles, and constitutive by green triangles. The letters ‘I’ and ‘A’ represent IPTG and Arabinose, respectively. Relative errors for GFP fluorescence are from the standard deviation of three biological replicates.

We screened the performance of the RNA-only IFFL circuits under different combinations of inducer IPTG (I) and Arabinose (A) concentrations (Figure 3 and Figure S3). In the absence of the 3WJ trigger (Decoy), X activated Z without indirect inhibition via Y such that a high GFP expression was observed. In this case, a high IPTG level (I = 500 µM and 125 µM) was required but a relatively low Arabinose concentration would be sufficient for GFP expression. When a regular Y was used, the GFP signal was observed throughout the experiment but at reduced levels, as compared to cases with the Decoy. Both the insulated and the constitutive Y nodes reduced GFP signals to very low levels for all inducer combinations. Together, we observed the expected decrease in GFP signals from regular Y to insulated Y and to constitutive triggers, in the order of repressor strengths. However, no clear pulse generation was observed for the combinations of inducers and inhibition strengths of node Y tested. We note that GFP expression did not necessarily increase monotonically as the inducer concentrations increased, possibly due to the saturation of promoter activity and cellular burden. Additionally, the GFP signal can be unreliable once cells reach a stationary phase (gray dashed area). Considering that an RNA trigger expressed from Y can bind to the RNA switch domain expressed from Z with fast kinetics, there may not be enough delays for the GFP reporter accumulation before inhibition becomes apparent. To confirm these speculations, we then explored the dynamics of RNA-only IFFL circuits through mathematical modeling, based on the experimental findings.

### 3.2. RNA-Only IFFL Modeling

We observe in all the experiments performed so far that no pulse in the GFP concentration was detected, and this contradicts the expectation for a typical IFFL circuit. To understand if this phenomenon was due to the limited range of inducer concentrations tested or it was because of the RNA-only IFFL circuit design itself, we constructed a mechanistic mathematical model to represent the key reactions in the design given in Figure 2a, leaving out the detailed STAR–RNA interaction from the model. Based on our experimental observation (Figure 3), the transcription induction by both IPTG and Arabinose indicated a nonlinear behavior. Therefore, we modeled the activity of IPTG and Arabinose using a Hill activation function. With that the ODEs are given by:

(1) $$\frac{d\left[X\right]}{dt}=\alpha_{X}\left[P_{X,total}\right]\frac{{\left[I_{ara}\right]}^{m}}{K_{ara}^{m}+{\left[I_{ara}\right]}^{m}}-\gamma \left[X\right]\left[P_{Y}^{*}\right]-\gamma \left[X\right]\left[P_{Z}^{*}\right]-\delta_{X}\left[X\right]$$

(2) $$\frac{d\left[Y\right]}{dt}=\alpha_{Y}\gamma \left[X\right]\left[P_{Y}^{*}\right]-\delta_{Y}\left[Y\right]-\omega \left[Y\right]\left[Z\right]$$

(3) $$\frac{d\left[Z\right]}{dt}=\alpha_{Z}\gamma \left[X\right]\left[P_{Z}^{*}\right]-\delta_{Z}\left[Z\right]-\omega \left[Y\right]\left[Z\right]$$

(4) $$\frac{d\left[GFP\right]}{dt}=\alpha_{GFP}\left[Z\right]-\delta_{GFP}\left[GFP\right]$$

(5) $$\left[P_{Y}^{*}\right]=\left[P_{Y,total}\right]\frac{{\left[I_{IPTG}\right]}^{n}}{K_{IPTG}^{n}+{\left[I_{IPTG}\right]}^{n}}$$

(6) $$\left[P_{Z}^{*}\right]=\left[P_{Z,total}\right]\frac{{\left[I_{IPTG}\right]}^{n}}{K_{IPTG}^{n}+{\left[I_{IPTG}\right]}^{n}}\;$$

where *α* is the production rate, *γ* and *ω* are the binding rates, and *δ* is the degradation rate. *I_ara_* and *I_IPTG_* are the Arabinose and IPTG inducers, respectively, *K_ara_* and *K_IPTG_* are the Hill constants, *m* and *n* are the Hill coefficients, $P_{X,total}$, $P_{Y,total}$ and $P_{Z,total}$ are the total plasmid concentrations for X, Y and Z. $P_{Y}^{*}$ and $P_{Z}^{*}$ are, respectively, the IPTG-induced Y and Z plasmids that are ready for transcription activation.

Model parameterization was performed by fitting to the experimental measurements with MATLAB function *fminsearch*, using the Nelder–Mead simplex algorithm [39]. The GFP fluorescence divided by the optical density of the cells observed experimentally was correlated with intracellular GFP concentration; however, we could not directly ascertain absolute concentration levels without calibration curves. Thus, we introduce a scaling factor $S_{G}$ to account for the potential magnitude differences. Specifically, experimental data with IPTG concentrations of 500 μM and 7.81 μM and Arabinose concentrations of 6600 μM, 1650 μM and 103.1 μM were used to fit a total of 15 parameters in the model. The fitted parameter values are summarized in Table S1, and the comparison of the fitted and the experimental GFP concentration is given in the top row of Figure S4.

With the fitted model, we first validated the prediction capability of the model with additional experiments with varying Arabinose and IPTG concentrations. The middle row of Figure S4 shows the comparison of the model prediction and the experimental measurement for different combinations of Arabinose and IPTG concentrations. A complete validation of the model is given in Figure S5. According to the comparison, we can see that the model was able to capture reasonably well the dynamics of the system for different Arabinose and IPTG concentrations.

Next, we used the model to predict the system dynamics under a wider range of IPTG and Arabinose concentrations. The results in the bottom row of Figures S4 and S6 indicate no pulse could be achieved with the RNA-only IFFL circuit, even if a wide range of inducer concentrations was allowed. Taken together, we speculated that the time delay of the inhibition might not have been sufficient to generate a pulse in the output GFP concentration, given the fact that both the activation and inhibition occurred at the RNA level, and a circuit with a significant timescale difference between the activation and the inhibition would be needed.

### 3.3. RNA–Protein Hybrid IFFL Modeling

Following the analysis of the RNA-only IFFL circuit, we then propose an RNA–protein hybrid IFFL circuit, which featured a THS for RNA-based translational activation and a transcription factor for the protein-based transcriptional repression. Such an RNA–protein hybrid design could be realized by introducing TetR as an intermediate molecule that accomplished the repression of the target gene.

**Figure 4:**
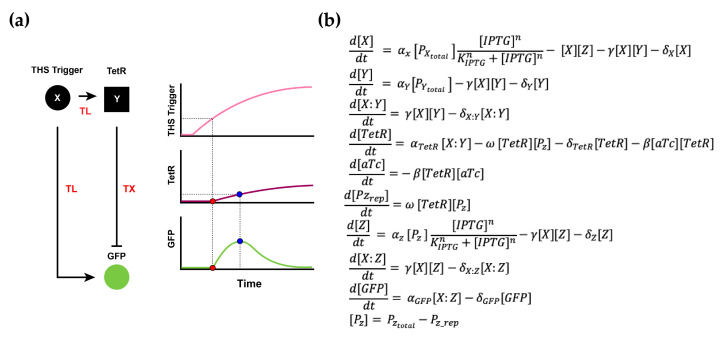
RNA–protein hybrid IFFL design and mathematical model. (**a**) The pulse generator design was implemented by fast THS activation and slow TetR repression. THS trigger activates GFP expression immediately while delayed TetR formation causes a pulse in the rate of GFP production. (**b**) Mathematical model for the RNA–protein hybrid circuit. Note, TX stands for transcription and TL stands for translation in the diagram. The red dot in the diagram indicates the time delay between the production of THS trigger from X and activation of Y and Z expression, and the blue dot indicates when the inhibition by TetR has an effect on GFP expression for a pulse to emerge in the system.

Our hypothesis was that the inclusion of the protein (TetR) formation would contribute to a sufficient amount of time delay in the inhibition pathway and would lead to a pulse generation in the output GFP concentration (Figure 4a). To further control the effect of the time delay of the inhibition pathway in the IFFL setup, we also introduced a nonlinear inhibition regulation on TetR via the addition of a titratable inducer molecule aTc.

We developed a mathematical model for the RNA–protein hybrid IFFL circuit, following the same argument as for the RNA-only IFFL circuit model development to validate our hypothesis (Figure 4b). X was the trigger RNA which, once bound to THS Y or Z, would allow the translation and eventually form the proteins TetR and GFP. This THS regulation mechanism was modeled with intermediate species X:Z and X:Y to account for the interactions between the trigger RNA (X) and the THS (Z and Y). The Hill activation function was used to account for the nonlinear effect of the inducer molecule IPTG on X and Z plasmids, and a simple inhibition reaction was used to simulate the repression of aTc on TetR. *α* is the production rate, *δ* is the degradation rate, *γ* and *ω* are the binding rates, $K_{IPTG}$ is the Hill constant, and *n* is the Hill coefficient. Z and Y are the transcribed THS RNAs that were activated by X to form the complexes X:Y and X:Z at the binding rate *γ*. $P_{X}$, $P_{Y}$ and $P_{Z}$ are the plasmids that transcribe X, Y, and Z at rate *α*, respectively. $P_{Zrep}$ is the repressed state of $P_{Z}$ that binds with TetR at rate *ω*.

**Figure 5:**
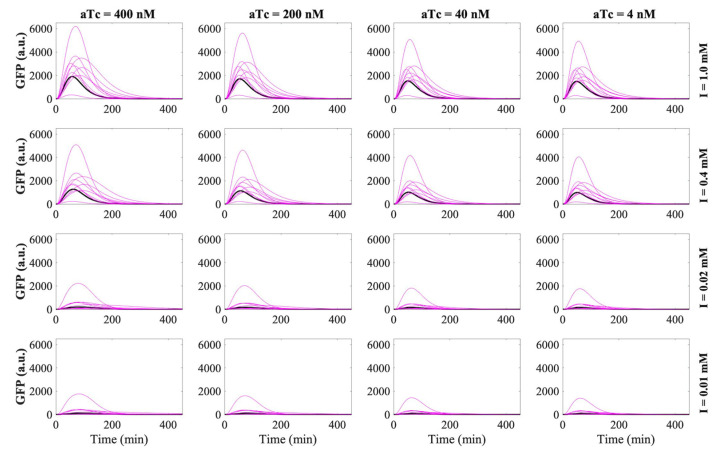
RNA–protein hybrid IFFL circuit simulation and kinetic parameter sensitivity analysis. The solid black line represents the model prediction at the nominal kinetic parameter estimates. The solid magenta line represents the ten random kinetic perturbations for each subplot. The letter ‘I’ represents IPTG.

Given the similarities between the RNA-only and the RNA–protein hybrid model, we estimated the RNA-related kinetic parameters in the hybrid model from the fitted values in the RNA-only model and inferred the protein-related kinetic parameters from literature to examine the dynamics of the proposed RNA–protein hybrid IFFL circuit. The detailed information regarding the parameter values are given in Table S2, and the GFP concentrations predicted with these parameters are plotted with the solid black lines for various combinations of inducer IPTG and aTc concentrations (Figure 5). Note, since we observed from the RNA-only IFFL circuit experiments that saturated IPTG levels can induce stress in the bacteria (Figure S3), we then confined the range of aTc and IPTG concentrations between 400 nM and 4 nM and 1 mM to 0.01 mM in our simulations, respectively.

Considering the possible deviations in the kinetic parameters due to changes in the design of the circuit and in the experimental conditions, we also allowed the kinetic parameters to vary within ±50% of the values identified in Table S2. By perturbing the parameters, we could have more confidence in the performance of the proposed RNA–protein hybrid IFFL design. As the purpose of this analysis was to confirm that a pulse can indeed be achieved even if the actual kinetic parameters deviate significantly from our estimation, a total of ten random perturbations were performed for each experimental condition and were plotted with the solid magenta lines. The detailed parameter sampling was described in the supporting information (Table S2). As shown in Figure 5, all the predictions demonstrated a pulse in the GFP concentration. As anticipated, changing the IPTG and aTc concentrations manipulated the pulse characteristics.

### 3.4. Experimental Realization and Validation of the RNA–Protein Hybrid IFFL Design

**Figure 6:**
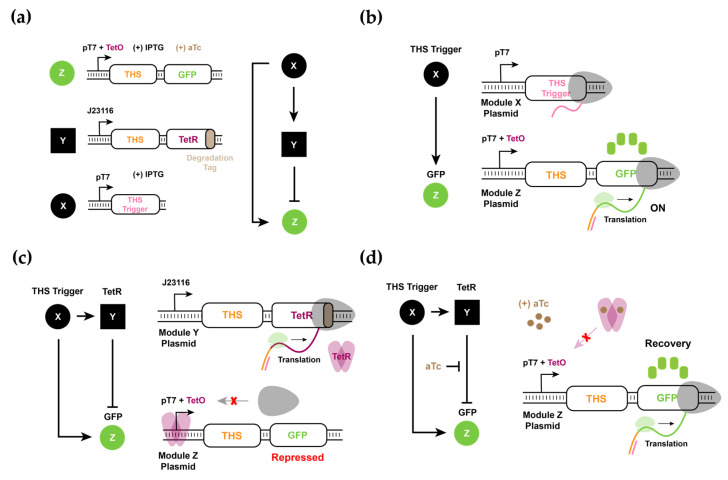
Architecture of RNA–protein hybrid IFFL circuit composed of THS and TetR regulation. (**a**) Detailed node constructs of RNA–protein hybrid IFFL circuit. Node Z contains the THS-GFP under the control of pT7 with TetO. Node Y contains the THS-TetR (with degradation tag) expressed under the constitutive promoter J23116. Node X contains the THS trigger expressed by pT7. (**b**) Schematic of the activation by X on Z. In the presence of the THS trigger, the start codon and RBS are exposed, thereby allowing translation of GFP protein. (**c**) Schematic of the inhibition by Y on Z. In the presence of THS trigger, TetR can be expressed, which binds to TetO within pT7 and blocks RNAP access to plasmid Z. In this case, the GFP expression will be repressed. (**d**) Schematic of aTc treatment in RNA–protein hybrid IFFL circuits. Even when the promoter is blocked by TetR, aTc treatment can release TetR from TetO within pT7 and GFP expression can be restored.

Encouraged by the modeling results, we then constructed and tested the RNA–protein hybrid circuit in vivo. The proposed RNA–protein hybrid circuit used a protein regulator, TetR, in the intermediate node Y to increase the time delay between the direct activation of Z from X and the indirect inhibition of Z from X via Y. The top regulatory node X contained a trigger RNA under the control of pT7. The intermediate node Y contained a THS followed by TetR under a constitutive promoter. The output node Z contained a THS followed by a GFP reporter under the control of pT7 that also contained a Tet operator site (TetO). These three nodes were encoded in separate plasmids such that different combinations of nodes could be easily explored (Figure 6a).

Experimentally, these plasmids were transformed in *E. coli* BL21 DE3 which harbors a genomic T7 RNAP under the control of Lac promoter. Thus, the expression levels of X and Z were controlled by IPTG, while the expression level of Y was constitutive. Further, the regulatory strength of node Y could be tuned as follows: changing the protein degradation tag on TetR, adjusting the promoter strength of Y, and the addition of a small molecule inducer aTc which titrates TetR. Since TetR can tightly bind to TetO within the promoter sequence, typically a very low concentration of TetR is sufficient to reduce the target promoter activity [28]. Thus, we chose a weak promoter (J23116) for the intermediate node Y that expressed THS followed by TetR. We also tested three different degradation tags with different degradation strengths (ASV, AAV, and LVA) attached to the C-terminus of TetR [40]. As the protease activity was shown to increase in the order of ASV, AAV, and LVA tags, TetR with a stronger degradation tag would likely have a weaker inhibition on GFP expression in the IFFL circuit. Further, aTc was added as a small molecule inducer to titrate the leaky TetR expression and to tune the time required to build up enough active TetR repressors.

To test the performance of THS and trigger RNA pairs, we first designed an activation experiment with input node X and output node Z. Upon the production of trigger RNA from X, the translation of GFP reporter from the transcript of Z would be initiated (Figure 6b). Since Z contained THS linked to the GFP reporter, and the TetR protein was not present in the system, the GFP fluorescence increased with the increasing concentration of inducer IPTG as expected. Note that at very high inducer levels, the response of the molecular circuit saturated and no further increase in GFP fluorescence outputs was observed.

Next, we included all three plasmids in *E. coli* and tested the behavior of the circuit with different inducer concentrations. The trigger RNA from X activated the translation of THS in Y and Z to translate TetR and GFP, respectively. The regulator protein, TetR, in turn, could inhibit the transcription of node Z by binding to the TetO in pT7 (Figure 6c). In this scenario, we expected that a pulse generation could be observed for certain combinations of node Y elements and aTc concentrations, presumably due to the increased delay between the direct activation of Z by X and the indirect inhibition of Z by X via Y (Figure 6d).

**Figure 7:**
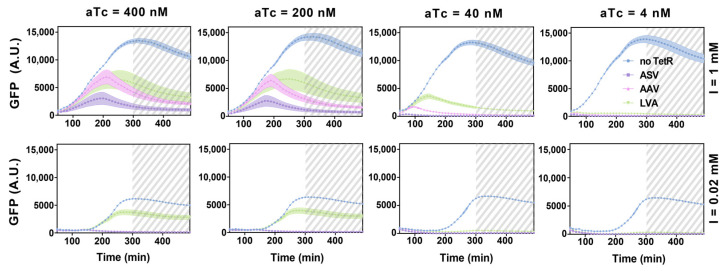
Experimental results for RNA–protein hybrid circuit. Time course measurement of GFP fluorescence for different inducer concentrations; IPTG concentrations at 1 mM and 0.02 mM; aTc concentrations at 400 nM, 200 nM, 40 nM and 4 nM. Y is represented by different symbols; no TetR by blue circles, TetR-ASV by purple squares, TetR-AAV by pink triangles, and TetR-LVA by green triangles. Data for the first 50 min were removed due to low OD600 values, and the time points beyond 300 min are marked as gray dashed area to indicate the transition to stationary phase. The letter ‘I’ represents IPTG. Relative errors for GFP fluorescence are from the s.d. of six biological replicates.

Analogous to the experiments performed for the RNA-only IFFL circuit, all three plasmids were simultaneously transformed into the *E. coli* BL21 DE3 strain and GFP fluorescence was measured to analyze the dynamics of the system. A wide range of IPTG and aTc concentrations were used to explore the response of circuits (Figure 7). Intriguingly, the experimental results showed a pronounced pulse for several inducer conditions, thereby confirming our modeling results that a pulse generation is possible for the hybrid circuit that shows an increased delay between direct activation and indirect inhibition. Experimental results showed a delayed pulse generation as compared to the simulation results in Figure 5, which could be due to the time it took to build up the T7 RNAP required for the transcription of node X and Z upon inducer treatment [17,19]. Notwithstanding the differences, the mathematical model can be trained with a selection of experimental data and reproduce a wider range of outcomes with only minor adjustments in parameter values (Figure S7, Table S3). Pulse generation was especially clear with high aTc and high IPTG concentrations. On the other hand, a low concentration of aTc correlated with a small pulse in GFP expression in line with the expected earlier onset of repression on Z by accumulated TetR. The degradation tags on TetR also showed expected trends where a stronger degradation tag correlated with a larger and extended pulse shape. Further experimental conditions are shown in Figure S8. We note that, unlike the dynamics of the RNA-only IFFL circuit, the RNA–protein hybrid IFFL circuit showed a clear decay in GFP signals hours before the cells entered the stationary phase (gray dashed area). Together, we verified that the RNA–protein hybrid IFFL circuit could show pulse generation in vivo, and that the results correlated well with the experimentally tunable parameters in the synthetic circuit.

## 4. Discussion

In this study, we utilized a number of de novo-designed RNA regulators, STAR, THS, and 3WJ repressors, to construct molecular circuits in vivo and explored their dynamic behaviors. A combination of a STAR and 3WJ repressor was used to construct the RNA-only IFFL circuit. The fast activation and repression kinetics of both RNA regulators demonstrated the expected system dynamics; however, there was no significant delay between the direct activation and the indirect inhibition of the final gene expression. These findings were confirmed in mechanistic modeling and simulation results. To increase delays between the direct activation and the indirect inhibition, we explored the RNA–protein hybrid regulatory model. The simulations showed that pulse generation could be achieved with this RNA–protein hybrid model as an IFFL circuit, which was further verified with experimental realization in *E. coli*. Together, the mechanistic understanding of de novo-designed RNA regulators and design flexibility allowed us to construct and validate different IFFL designs and achieve a pulse generation through controlling the regulation timescale differences in *E. coli*.

A number of synthetic IFFLs have been demonstrated using protein and RNA regulators. The synthetic protein regulator-based IFFL circuits are typically composed of well-known transcription regulators [34,41,42], and the synthetic RNA-based IFFL circuits are normally achieved with STAR and dCas9-based transcription regulation [25,37]. Here, we constructed two simple networks that implement an IFFL network motif with a novel combination of RNA regulators. The first network implemented a synthetic circuit with STAR and 3WJ repressors, establishing the RNA-only IFFL circuit. We further constructed the RNA–protein hybrid IFFL circuit with THS as the trigger for activation and repression. These synthetic circuits demonstrated distinct dynamical features and provided interesting case studies for constructing dynamical circuits in vivo. Indeed, the failure of the RNA-only IFFL circuit in generating a pulse in the target gene expression manifested that while RNA-based regulators have been widely acclaimed for rapid signal propagation, such a fast reaction kinetic associated with RNA–RNA interactions might make it challenging to achieve certain dynamics when an RNA-only circuit is designed. This observation further indicates that when using RNA-based regulators in a timescale-critical circuit, tuning of the components might be needed, and to facilitate the design and tuning, modeling can then be leveraged, as illustrated in this study.

The RNA-only IFFL circuit model was parameterized with experimental measurements, to carry out a comprehensive analysis of the dynamics in simulation. The simulated results were found to correlate well with our experimental findings, and further supported the hypothesis that the lack of a sufficient time delay in the inhibition pathway hampered the generation of a pulse in the target gene. Simulations with the RNA–protein hybrid model demonstrated that with the similar parameter values from the RNA-only model, the addition of the protein synthesis did help achieve the pulse generation, which was confirmed with experiments in vivo. These findings indicate the reliability of the well-constructed and parameterized model in guiding the design of experiments for more complex synthetic molecular systems.

The modularity and composability of synthetic molecular parts are important requirements for the development of complex synthetic biological circuits. We showed that the de novo synthetic RNA regulators STAR and THS (both for activators and repressors) can be flexibly combined to achieve desired dynamics. Previous works have shown that STAR and THS can be combined to form AND-gate circuits [43,44], and here we presented for the first time the combination of STAR and 3WJ repressors in the design of a genetic circuit. Moreover, we also showed that THS can be used to regulate circuit dynamics beyond the currently reported combinatorial logic [18]. Combining a large library of STAR, THS and repressors together with a large library of protein regulators such as the TetR family transcription factors [45] could expand the repertoire of dynamical systems with the potential benefit of porting the modeling framework and parameters from the detailed characterization shown here.

In summary, we demonstrated the RNA-only circuit and the RNA–protein hybrid circuit for IFFL and explored their dynamics with both experiments and modeling. We demonstrated how mathematical modeling can be used to assess and guide the experimental design of synthetic biological circuits. We also found that the parameters obtained from the RNA-only IFFL circuit could also be applicable to the RNA–protein hybrid IFFL circuit, and this transferability indicates that the RNA regulator can be flexibly combined with other regulatory components without experiencing dramatic changes in the kinetics. Together, we demonstrated the utility of de novo-designed RNA regulators for synthetic circuits, and the combined use of mechanistic mathematical modeling in guiding and optimizing the design of synthetic circuits with diverse parts.

## Data Availability

The data presented in this study are available on request from the corresponding author.

## Supplementary Materials

The following are available online at https://www.mdpi.com/article/10.3390/biom11081182/s1, Table S1: estimated kinetic parameters for RNA-only IFFL circuit by fitting the model to the experimental GFP data, Table S2: RNA–protein hybrid IFFL circuit initial parameter estimation, Table S3: estimated kinetic parameters for RNA–protein hybrid circuit by fitting the model to the experimental data, Table S4: plasmids used in this study, Table S5: examples of RNA-only IFFL DNA plasmid sequences, Table S6: examples of RNA–protein hybrid IFFL DNA plasmid sequences, Table S7: promoter sequences used in this study, Table S8: insert sequences used in this study, Table S9: other Accessary sequences used in this study, Figure S1: in vivo characterization of the X to Z activation in the RNA-only IFFL circuit, Figure S2: RNA secondary structures for four different variants of Y in the RNA-only IFFL circuit, Figure S3: in vivo characterization of the RNA-only IFFL circuit, Figure S4: RNA-only IFFL experiment and simulation, Figure S5: RNA-only IFFL experiment and simulated GFP data for different IPTG and Arabinose concentrations, Figure S6: simulated RNA-only IFFL circuit for different combinations of IPTG and Arabinose concentrations, Figure S7: RNA–protein hybrid IFFL simulation and experimental GFP data, Figure S8: experimental results for RNA–protein hybrid circuit.
