# Supplementary material for: Design and Evaluation of Synthetic RNA-Based Incoherent Feed-Forward Loop Circuits
Source: Biomolecules. 2021 Aug 10;11(8):1182. doi: 10.3390/biom11081182 (PMC8391864; doi:10.3390/biom11081182)
Supplement: Supplementary file 1 [file biomolecules-11-01182-s001.zip › biomolecules-1300227-supplementary.pdf]

biomolecules

Design and Evaluation of Synthetic RNA-based Incoherent Feed-forward Loop Circuits

Supplementary Information

Seongho Hong [1,†], Dohyun Jeong [1,†], Jordan Ryan [2,†], Mathias Foo [3,*], Xun Tang [2,*] and Jongmin Kim [1,*]

1 Department of Life Sciences, Pohang University of Science and Technology, Pohang 37673, Korea; shhong1205@postech.ac.kr (S.H.); gyu9506@postech.ac.kr (D.J.)
2 Cain Department of Chemical Engineering, Louisiana State University, Baton Rouge, LA 70803, USA; jryan34@lsu.edu
3 School of Engineering, University of Warwick, Coventry CV4 7AL, UK
* Correspondence: M.Foo@warwick.ac.uk (M.F.); xuntang@lsu.edu (X.T.); jongmin.kim@postech.ac.kr (J.K.)
† Equal contribution: Seongho Hong, Dohyun Jeong, Jordan Ryan.

Detailed Experimental Protocols

Plasmid construction

Plasmids were constructed using PCR, Gibson assembly and round-the-horn site-directed mutagenesis. All DNA templates for RNA only IFFL and RNA-protein hybrid IFFL were assembled from single-stranded DNAs purchased from Bionics. RNA regulatory part sequences were selected as follows: STAR-target pair is AD1.A5-AD1.S5 [1]; THS-trigger pair is ACTS_TypeII_N3 [2]; 3WJ repressor-trigger pair is 3WJrep_N19 [3]. The synthetic DNA strands were amplified via PCR to form double-stranded DNAs. The resulting DNAs were then inserted into plasmid backbones using about 30-bp homology domains via Gibson assembly [4]. Promoter change from pT7 to other promoters (pLlacO, J23116, pT7(TetO)) and degradation tag addition were done by round-the-horn site-directed mutagenesis. All plasmids were cloned in the *E.coli* DH5a strain and validated through DNA Sequencing. Backbones for the plasmids were taken from the commercial vectors pET15b, pCDFDuet, pCOLADuet (EMD Millipore). GFPmut3b-ASV was used as the reporter. This GFP is GFPmut3b with an ASV degradation tag [5]. TetR was used with three different degradation tags (ASV, AAV and LVA). Sequences of elements commonly used in the plasmids are provided in Table S5–S9.

Supplementary Tables

Supplementary Table S1. Estimated kinetic parameters for RNA-only IFFL circuit by fitting the model to the experimental GFP data.

Parameter	Value	Unit
α_X	1.5630	sec^{-1}
α_Y	0.9238	sec^{-1}
α_Z	0.0032	sec^{-1}
α_{GFP}	0.0202	sec^{-1}
δ_X	0.0005	sec^{-1}
δ_Y	0.0725	sec^{-1}
δ_Z	0.0063	sec^{-1}
δ_{GFP}	0.0002	sec^{-1}
γ	11186.9458	$M^{-1}sec^{-1}$
ω	5602.5417	$M^{-1}sec^{-1}$
K_{ara}	0.0002	M
K_{IPTG}	0.0015	M
S_G	4.4462×10^{11}	-
m	0.0453	-
n	0.1850	-

Model parameterization for the RNA-only IFFL circuit is performed by fitting to the experimental GFP data using MATLAB function fminsearch, that uses the Nelder-Mead simplex algorithm [6]. Since the GFP concentration is represented as the optical density of the cells, we introduce a scaling factor S_G to account for the potential magnitude differences. The experimental GFP data with IPTG concentrations of 500 μM and 7.81 μM and Arabinose concentrations of 6600 μM, 1650 μM and 103.125 μM are used to fit a total of 15 parameters in the model. The fitted parameter values are shown in Table S1, and the comparison of the fitted and the experimental GFP data is given in the top row of Figure 3 in the main text. Here, we want to remark that the estimated Hill coefficients m and n are less than unity, which are somewhat different from the values found in literature [7,8]. Hill coefficient that is less than unity indicates negative cooperativity [9]. Based on our data availability, we do observe this negative cooperativity trend in our experimental GFP data that is consistent with our estimated values of m and n.

Supplementary Table S2. RNA-protein hybrid IFFL circuit initial parameter estimation. Parameter values derived from RNA-only IFFL circuit estimation were rounded to generalize the parameter estimation for the hybrid circuit. The Lower and Upper bound columns denote the bounds used for the sensitivity analysis featured in Figure 5 in the main text.

Parameter	Value	Lower Bound (-50%)	Upper Bound (+50%)	Unit
α_X	1.0000	0.5000	1.5000	sec^{-1}
α_Y	1.0000	0.5000	1.5000	sec^{-1}
α_Z	0.0500	0.0250	0.0750	sec^{-1}
δ_X	0.0050	0.0025	0.0075	sec^{-1}
δ_Y	0.0500	0.0250	0.0750	sec^{-1}
δ_Z	0.0050	0.0025	0.0075	sec^{-1}
α_{TetR}	0.5000	0.2500	0.7500	sec^{-1}
δ_{TetR}	0.0005	0.00025	0.00075	sec^{-1}
α_{GFP}	0.5000	0.2500	0.7500	sec^{-1}
δ_{GFP}	0.0005	0.00025	0.00075	sec^{-1}
ω	5000	2500	7500	$M^{-1}sec^{-1}$
γ	10000	5000	15000	$M^{-1}sec^{-1}$
$\delta_{X:Z}$	0.0010	0.0005	0.0015	sec^{-1}
$\delta_{X:Y}$	0.0010	0.0005	0.0015	sec^{-1}
S_{Exp}	10	-	-	-
n	0.5000	0.2500	0.7500	-
K_{IPTG}	0.0010	0.0005	0.0015	M
β	10000	5000	15000	$M^{-1}sec^{-1}$

Table S2 summarizes the parameters estimated from the RNA-only model parameters given in Table S1, to use in the RNA-protein hybrid model to investigate the feasibility of generating a pulse in the GFP concentration. The parameter S_{Exp} is a scaling factor and performs a similar function to the scaling factor in the RNA-only model; however, the parameter is used as an exponential to scale the output rather than scalar multiplier as used previously. The parameters are varied with a ±50% change to the nominal values, to perform a sensitivity analysis on the circuit dynamics, to account for effects from changing design and experimental setup.

Supplementary Table S3. Estimated kinetic parameters for RNA-protein hybrid circuit by fitting the model to the experimental data.

Parameter	Value	Unit
α_X	1.8170	sec^{-1}
α_Y	0.7270	sec^{-1}
α_Z	0.1570	sec^{-1}
δ_X	0.0330	sec^{-1}
δ_Y	0.0920	sec^{-1}
δ_Z	0.0090	sec^{-1}
α_{TetR}	0.2030	sec^{-1}
δ_{TetR}	0.0001	sec^{-1}
α_{GFP}	0.0065	sec^{-1}
δ_{GFP}	0.0025	sec^{-1}
ω	5000	$M^{-1}sec^{-1}$
γ	10000	$M^{-1}sec^{-1}$
$\delta_{X:Z}$	0.0002	sec^{-1}

$\delta_{X:Y}$	0.0056	sec^{-1}
S_{Exp}	8.0	-
n	0.3370	-
K_{IPTG}	1.51×10^{-4}	M
β	10000	$M^{-1}sec^{-1}$

Table S3 summarizes the kinetic parameters estimated for the RNA-protein hybrid IFFL circuit by fitting the model to the experimental data in Figure 7 in the main text. Comparing the values in Tables S1 and S3, we notice there are a few similarities such as the relative α or transcription value are slightly higher for the Y. Additionally, the γ and ω values stay relatively constant for both models. Notably, the dissociation constant for IPTG is lower for the hybrid circuit; nonetheless, the RNA-only circuit parameters generally translate to the RNA-protein hybrid circuit.

Supplementary Table S4. Plasmids used in this study. Abbreviations are as follows: T7term = T7 terminator, AmpR = ampicillin resistance gene, SpecR = spectinomycin resistance gene, KanR = kanamycin resistance gene, CmR = chloramphenicol resistance gene.

Name	Plasmid Architecture
RNA only IFFL circuit	
X	pT7–STAR–T7term–AmpR–pBR322 origin
Y	pLlacO–STAR Target–3WJ Trigger–T7term–SpecR–CloDF13 origin
Insulated Y	pLlacO–STAR Target–RiboJ–3WJ Trigger–T7term–SpecR–CloDF13 origin
Decoy	pLlacO–Decoy–T7term–SpecR–CloDF13 origin
Constitutive	pLlacO–3WJ Trigger–T7term–SpecR–CloDF13 origin
Z	pLlacO–STAR Target –3WJ Repressor–Linker–GFPmut3b-ASV–T7term–KanR–ColA origin
RNA-protein hybrid IFFL circuit	
X	pT7–THS Trigger–T7term–AmpR–pBR322 origin
Y_No TetR	J23116–Linker–TetR–T7term–SpecR–CloDF13 origin
Y_ASV tagged	J23116–THS–Linker–TetR–ASV–T7term–SpecR–CloDF13 origin
Y_AAV tagged	J23116–THS–Linker–TetR–AAV–T7term–SpecR–CloDF13 origin
Y_LVA tagged	J23116–THS–Linker–TetR–LVA–T7term–SpecR–CloDF13 origin
Z	pT7–TetO–THS–Linker–GFPmut3b-ASV–T7term–KanR–ColA origin

Supplementary Table S5. Examples of **RNA only IFFL** DNA plasmid sequences.

Name (architecture)	Sequence
X (pT7–STAR–T7term–(Bla Promoter)–AmpR–pBR322 origin–backbone)	TAATACGACTCACTATAGGGTGAACTGTATACATTCCCCGCTGCTCCAACA<br>TTTATACAACTAATTAAAACAATTCACTGTAAAAACTTAGCATAACCCCTT<br>GGGGCCTCTAAACGGGTCTTGAGGGGTTTTTTGCTGAAAGGAGGAACTATA<br>TCCGGATATCCCGCAAGAGGCCCGGCAGTACCGGCATAACCAAGCCTATG<br>CCTACAGCATCCAGGGTGACGGTGCCGAGGATGACGATGAGCGCATTGTT<br>AGATTTCATACACGGTGCCTGACTGCGTTAGCAATTTAACTGTGATAAACT<br>ACCGCATTAAAGCTTATCGATGATAAGCTGTCAAACATGAGAATTCTTGAA<br>GACGAAAGGGCCTCGTGATACGCCTATTTTTATAGGTTAATGTCATGATAA<br>TAATGGTTTCTTAGACGTCAGGTGGCACTTTTCGGGGAAATGTGCGCGGAA<br>CCCCTATTTGTTTATTTTTCTAAATACATTCAAATATGTATCCGCTCATGAGA<br>CAATAACCCTGATAAATGCTTCAATAATATTGAAAAAGGAAGAGTATGAG<br>TATTCAACATTTCCGTGTCGCCCTTATTCCCTTTTTTGCGGCATTTTGCCTTCC<br>TGTTTTTGCTCACCCAGAAACGCTGGTGAAAGTAAAAGATGCTGAAGATCA<br>GTTGGGTGCACGAGTGGGTTACATCGAACTGGATCTCAACAGCGGTAAGAT

	CCTTGAGAGTTTTCGCCCCGAAGAACGTTTTCCAATGATGAGCACTTTTAA<br>AGTTCTGCTATGTGGCGCGGTATTATCCCGTGTTGACGCCGGGCAAGAGCA<br>ACTCGGTCGCCGCATACACTATTCTCAGAATGACTTGGTTGAGTACTCACC<br>AGTCACAGAAAAGCATCTTACGGATGGCATGACAGTAAGAGAATTATGCA<br>GTGCTGCCATAACCATGAGTGATAACACTGCGGCCAACTTACTTCTGACAA<br>CGATCGGAGGACCGAAGGAGCTAACCGCTTTTTTGCACAACATGGGGGAT<br>CATGTAACTCGCCTTGATCGTTGGGAACCGGAGCTGAATGAAGCCATACCA<br>AACGACGAGCGTGACACCACGATGCCTGCAGCAATGGCAACAACGTTGCG<br>CAAACTATTAACTGGCGAACTACTTACTCTAGCTTCCCGGCAACAATTAAT<br>AGACTGGATGGAGGCGGATAAAGTTGCAGGACCACTTCTGCGCTCGGCCC<br>TTCCGGCTGGCTGGTTTATTGCTGATAAATCTGGAGCCGGTGAGCGTGGGT<br>CTCGCGGTATCATTGCAGCACTGGGGCCAGATGGTAAGCCCTCCCGTATCG<br>TAGTTATCTACACGACGGGGAGTCAGGCAACTATGGATGAACGAAATAGA<br>CAGATCGCTGAGATAGGTGCCTCACTGATTAAGCATTGGTAACTGTCAGAC<br>CAAGTTTACTCATATATACTTTAGATTGATTTAAAACTTCATTTTTAATTTAA<br>AAGGATCTAGGTGAAGATCCTTTTTGATAATCTCATGACCAAAATCCCTTA<br>ACGTGAGTTTTCGTTCCACTGAGCGTCAGACCCCGTAGAAAAGATCAAAG<br>GATCTTCTTGAGATCCTTTTTTTCTGCGCGTAATCTGCTGCTTGCAAACAAA<br>AAAACCACCGCTACCAGCGGTGGTTTGTTTGCCGGATCAAGAGCTACCAAC<br>TCTTTTTCCGAAGGTAACTGGCTTCAGCAGAGCGCAGATACCAAATACTGT<br>CCTTCTAGTGTAGCCGTAGTTAGGCCACCACTTCAAGAACTCTGTAGCACC<br>GCCTACATACCTCGCTCTGCTAATCCTGTTACCAGTGGCTGCTGCCAGTGGC<br>GATAAGTCGTGTCTTACCGGGTTGGACTCAAGACGATAGTTACCGGATAAG<br>GCGCAGCGGTCGGGCTGAACGGGGGGTTCGTGCACACAGCCCAGCTTGGA<br>GCGAACGACCTACACCGAACTGAGATACCTACAGCGTGAGCTATGAGAAA<br>GCGCCACGCTTCCCGAAGGGAGAAAGGCGGACAGGTATCCGGTAAGCGGC<br>AGGGTCGGAACAGGAGAGCGCACGAGGGAGCTTCCAGGGGGAAACGCCT<br>GGTATCTTTATAGTCCTGTCGGGTTTCGCCACCTCTGACTTGAGCGTCGATT<br>TTTGTGATGCTCGTCAGGGGGGCGGAGCCTATGGAAAAACGCCAGCAACG<br>CGGCCTTTTTACGGTTCCTGGCCTTTTGCTGGCCTTTTGCTCACATGTTCTTTC<br>CTGCGTTATCCCCTGATTCTGTGGATAACCGTATTACCGCCTTTGAGTGAGC<br>TGATACCGCTCGCCGCAGCCGAACGACCGAGCGCAGCGAGTCAGTGAGCG<br>AGGAAGCGGAAGAGCGCCTGATGCGGTATTTTCTCCTTACGCATCTGTGCG<br>GTATTTCACACCGCATATATGGTGCACTCTCAGTACAATCTGCTCTGATGCC<br>GCATAGTTAAGCCAGTATACACTCCGCTATCGCTACGTGACTGGGTCATGG<br>CTGCGCCCCGACACCCGCCAACACCCGCTGACGCGCCCTGACGGGCTTGTC<br>TGCTCCCGGCATCCGCTTACAGACAAGCTGTGACCGTCTCCGGGAGCTGCA<br>TGTGTCAGAGGTTTTCACCGTCATCACCGAAACGCGCGAGGCAGCTGCGGT<br>AAAGCTCATCAGCGTGGTCGTGAAGCGATTCACAGATGTCTGCCTGTTCAT<br>CCGCGTCCAGCTCGTTGAGTTTCTCCAGAAGCGTTAATGTCTGGCTTCTGAT<br>AAAGCGGGCCATGTTAAGGGCGGTTTTTTCCTGTTTGGTCACTGATGCCTCC<br>GTGTAAGGGGGATTTCTGTTCATGGGGGTAATGATACCGATGAAACGAGA<br>GAGGATGCTCACGATACGGGTTACTGATGATGAACATGCCCGGTTACTGGA<br>ACGTTGTGAGGGTAAACAACTGGCGGTATGGATGCGGCGGGACCAGAGAA<br>AAATCACTCAGGGTCAATGCCAGCGCTTCGTTAATACAGATGTAGGTGTTC<br>CACAGGGTAGCCAGCAGCATCCTGCGATGCAGATCCGGAACATAATGGTG<br>CAGGGCGCTGACTTCCGCGTTTCCAGACTTTACGAAACACGGAAACCGAA<br>GACCATTCATGTTGTTGCTCAGGTCGCAGACGTTTTGCAGCAGCAGTCGCTT<br>CACGTTCGCTCGCGTATCGGTGATTCATTCTGCTAACCAGTAAGGCAACCC<br>CGCCAGCCTAGCCGGGTCCTCAACGACAGGAGCACGATCATGCGCACCCG<br>TGGCCAGGACCCAACGCTGCCCGAGATGCGCCGCGTGCGGCTGCTGGAGA

	TGGCGGACGCGATGGATATGTTCTGCCAAGGGTTGGTTTGCGCATTCACAG<br>TTCTCCGCAAGAATTGATTGGCTCCAATTCTTGGAGTGGTGAATCCGTTAGC<br>GAGGTGCCGCCGGCTTCCATTCAGGTCGAGGTGGCCCGGCTCCATGCACCG<br>CGACGCAACGCGGGGAGGCAGACAAGGTATAGGGCGGCGCCTACAATCC<br>ATGCCAACCCGTTCCATGTGCTCGCCGAGGCGGCATAAATCGCCGTGACGA<br>TCAGCGGTCCAGTGATCGAAGTTAGGCTGGTAAGAGCCGCGAGCGATCCTT<br>GAAGCTGTCCCTGATGGTCGTCATCTACCTGCCTGGACAGCATGGCCTGCA<br>ACGCGGGCATCCCGATGCCGCCGGAAGCGAGAAGAATCATAATGGGGAA<br>GGCCATCCAGCCTCGCGTCGCGAACGCCAGCAAGACGTAGCCCAGCGCGT<br>CGGCCGCCATGCCGGCGATAATGGCCTGCTTCTCGCCGAAACGTTTGGTGG<br>CGGGACCAGTGACGAAGGCTTGAGCGAGGGCGTGCAAGATTCCGAATACC<br>GCAAGCGACAGGCCGATCATCGTCGCGCTCCAGCGAAAGCGGTCCTCGCC<br>GAAAATGACCCAGAGCGCTGCCGGCACCTGTCCTACGAGTTGCATGATAA<br>AGAAGACAGTCATAAGTGCGGCGACGATAGTCATGCCCCGCGCCCACCGG<br>AAGGAGCTGACTGGGTTGAAGGCTCTCAAGGGCATCGGTCGAGATCCCGG<br>TGCCTAATGAGTGAGCTAACTTACATTAATTGCGTTGCGCTCACTGCCCGCT<br>TTCCAGTCGGGAAACCTGTCGTGCCAGCTGCATTAATGAATCGGCCAACGC<br>GCGGGGAGAGGCGGTTTGCGTATTGGGCGCCAGGGTGGTTTTTCTTTTCACC<br>AGTGAGACGGGCAACAGCTGATTGCCCTTCACCGCCTGGCCCTGAGAGAG<br>TTGCAGCAAGCGGTCCACGCTGGTTTGCCCCAGCAGGCGAAAATCCTGTTT<br>GATGGTGGTTAACGGCGGGATATAACATGAGCTGTCTTCGGTATCGTCGTA<br>TCCCACTACCGAGATATCCGCACCAACGCGCAGCCCGGACTCGGTAATGG<br>CGCGCATTGCGCCCAGCGCCATCTGATCGTTGGCAACCAGCATCGCAGTGG<br>GAACGATGCCCTCATTCAGCATTTGCATGGTTTGTTGAAAACCGGACATGG<br>CACTCCAGTCGCCTTCCCGTTCCGCTATCGGCTGAATTTGATTGCGAGTGAG<br>ATATTTATGCCAGCCAGCCAGACGCAGACGCGCCGAGACAGAACTTAATG<br>GGCCCGCTAACAGCGCGATTTGCTGGTGACCCAATGCGACCAGATGCTCCA<br>CGCCCAGTCGCGTACCGTCTTCATGGGAGAAAATAATACTGTTGATGGGTG<br>TCTGGTCAGAGACATCAAGAAATAACGCCGGAACATTAGTGCAGGCAGCT<br>TCCACAGCAATGGCATCCTGGTCATCCAGCGGATAGTTAATGATCAGCCCA<br>CTGACGCGTTGCGCGAGAAGATTGTGCACCGCCGCTTTACAGGCTTCGACG<br>CCGCTTCGTTCTACCATCGACACCACCACGCTGGCACCCAGTTGATCGGCG<br>CGAGATTTAATCGCCGCGACAATTTGCGACGGCGCGTGCAGGGCCAGACT<br>GGAGGTGGCAACGCCAATCAGCAACGACTGTTTGCCCGCCAGTTGTTGTGC<br>CACGCGGTTGGGAATGTAATTCAGCTCCGCCATCGCCGCTTCCACTTTTTCC<br>CGCGTTTTCGCAGAAACGTGGCTGGCCTGGTTCACCACGCGGGAAACGGTC<br>TGATAAGAGACACCGGCATACTCTGCGACATCGTATAACGTTACTGGTTTC<br>ACATTCACCACCCTGAATTGACTCTCTTCCGGGCGCTATCATGCCATACCGC<br>GAAAGGTTTTGCGCCATTCGATGGTGTCCGGGATCTCGACGCTCTCCCTTAT<br>GAACGTGTACGGGCTATCTGGCTTTCGTTGCGC
Y (pLlacO–STAR Target–3WJ Trigger–T7term–SpecR–(Bla Promoter)–CloDF13 origin–backbone)	ATAAATGTGAGCGGATAACATTGACATTGTGAGCGGATAACAAGATACTG<br>AGCACGGAGTTTTACAGTGAATTGTTTTAATTAGTTGTATAAATGTTGGAG<br>CAGCGGGGAATGTATACAGTTCATGTATATATTCCCCGCTTTTTTTTTGGGA<br>CCTAACATAAACTTGTTAGGTGCGTAGATCTGATTAGTGTGACCTAGCATA<br>ACCCCTTGGGGCCTCTAAACGGGTCTTGAGGGGTTTTTTGCTGAAACCTCA<br>GGCATTTGAGAAGCACACGGTCACACTGCTTCCGGTAGTCAATAAACCGGT<br>AAACCAGCAATAGACATAAGCGGCTATTTAACGACCCTGCCCTGAACCGA<br>CGACCGGGTCATCGTGGCCGGATCTTGCGGCCCCTCGGCTTGAACGAATTG<br>TTAGACATTATTTGCCGACTACCTTGGTGATCTCGCCTTTCACGTAGTGGAC<br>AAATTCTTCCAACTGATCTGCGCGCGAGGCCAAGCGATCTTCTTCTTGTCCA<br>AGATAAGCCTGTCTAGCTTCAAGTATGACGGGCTGATACTGGGCCGGCAG

	GCGCTCCATTGCCCAGTCGGCAGCGACATCCTTCGGCGCGATTTTGCCGGT<br>TACTGCGCTGTACCAAATGCGGGACAACGTAAGCACTACATTTCGCTCATC<br>GCCAGCCCAGTCGGGCGGCGAGTTCCATAGCGTTAAGGTTTCATTTAGCGC<br>CTCAAATAGATCCTGTTCAGGAACCGGATCAAAGAGTTCCTCCGCCGCTGG<br>ACCTACCAAGGCAACGCTATGTTCTCTTGCTTTTGTCAGCAAGATAGCCAG<br>ATCAATGTCGATCGTGGCTGGCTCGAAGATACCTGCAAGAATGTCATTGCG<br>CTGCCATTCTCCAAATTGCAGTTCGCGCTTAGCTGGATAACGCCACGGAAT<br>GATGTCGTCGTGCACAACAATGGTGACTTCTACAGCGCGGAGAATCTCGCT<br>CTCTCCAGGGGAAGCCGAAGTTTCCAAAAGGTCGTTGATCAAAGCTCGCC<br>GCGTTGTTTCATCAAGCCTTACGGTCACCGTAACCAGCAAATCAATATCAC<br>TGTGTGGCTTCAGGCCGCCATCCACTGCGGAGCCGTACAAATGTACGGCCA<br>GCAACGTCGGTTCGAGATGGCGCTCGATGACGCCAACTACCTCTGATAGTT<br>GAGTCGATACTTCGGCGATCACCGCTTCCCTCATACTCTTCCTTTTTCAATAT<br>TATTGAAGCATTTATCAGGGTTATTGTCTCATGAGCGGATACATATTTGAAT<br>GTATTTAGAAAAATAAACAAATAGCTAGCTCACTCGGTCGCTACGCTCCGG<br>GCGTGAGACTGCGGCGGGCGCTGCGGACACATACAAAGTTACCCACAGAT<br>TCCGTGGATAAGCAGGGGACTAACATGTGAGGCAAAACAGCAGGGCCGC<br>GCCGGTGGCGTTTTTCCATAGGCTCCGCCCTCCTGCCAGAGTTCACATAAA<br>CAGACGCTTTTCCGGTGCATCTGTGGGAGCCGTGAGGCTCAACCATGAATC<br>TGACAGTACGGGCGAAACCCGACAGGACTTAAAGATCCCCACCGTTTCCG<br>GCGGGTCGCTCCCTCTTGCGCTCTCCTGTTCCGACCCTGCCGTTTACCGGAT<br>ACCTGTTCCGCCTTTCTCCCTTACGGGAAGTGTGGCGCTTTCTCATAGCTCA<br>CACACTGGTATCTCGGCTCGGTGTAGGTCGTTCGCTCCAAGCTGGGCTGTA<br>AGCAAGAACTCCCCGTTCAGCCCGACTGCTGCGCCTTATCCGGTAACTGTT<br>CACTTGAGTCCAACCCGGAAAAGCACGGTAAAACGCCACTGGCAGCAGCC<br>ATTGGTAACTGGGAGTTCGCAGAGGATTTGTTTAGCTAAACACGCGGTTGC<br>TCTTGAAGTGTGCGCCAAAGTCCGGCTACACTGGAAGGACAGATTTGGTTG<br>CTGTGCTCTGCGAAAGCCAGTTACCACGGTTAAGCAGTTCCCCAACTGACT<br>TAACCTTCGATCAAACCACCTCCCCAGGTGGTTTTTTCGTTTACAGGGCAAA<br>AGATTACGCGCAGAAAAAAAGGATCTCAAGAAGATCCTTTGATCTTTTCTA<br>CTGAACCGCTCTAGATTTCAGTGCAATTTATCTCTTCAAATGTAGCACCTGA<br>AGTCAGCCCCATACGATATAAGTTGTAATTCTCATGTTAGTCATGCCCCGC<br>GCCCACCGGAAGGAGCTGACTGGGTTGAAGGCTCTCAAGGGCATCGGTCG<br>AGATCCCGGTGCCTAATGAGTGAGCTAACTTACATTAATTGCGTTGCGCTC<br>ACTGCCCGCTTTCCAGTCGGGAAACCTGTCGTGCCAGCTGCATTAATGAAT<br>CGGCCAACGCGCGGGGAGAGGCGGTTTGCGTATTGGGCGCCAGGGTGGTT<br>TTTCTTTTCACCAGTGAGACGGGCAACAGCTGATTGCCCTTCACCGCCTGGC<br>CCTGAGAGAGTTGCAGCAAGCGGTCCACGCTGGTTTGCCCCAGCAGGCGA<br>AAATCCTGTTTGATGGTGGTTAACGGCGGGATATAACATGAGCTGTCTTCG<br>GTATCGTCGTATCCCACTACCGAGATGTCCGCACCAACGCGCAGCCCGGAC<br>TCGGTAATGGCGCGCATTGCGCCCAGCGCCATCTGATCGTTGGCAACCAGC<br>ATCGCAGTGGGAACGATGCCCTCATTCAGCATTTGCATGGTTTGTTGAAAA<br>CCGGACATGGCACTCCAGTCGCCTTCCCGTTCCGCTATCGGCTGAATTTGAT<br>TGCGAGTGAGATATTTATGCCAGCCAGCCAGACGCAGACGCGCCGAGACA<br>GAACTTAATGGGCCCGCTAACAGCGCGATTTGCTGGTGACCCAATGCGACC<br>AGATGCTCCACGCCCAGTCGCGTACCGTCTTCATGGGAGAAAATAATACTG<br>TTGATGGGTGTCTGGTCAGAGACATCAAGAAATAACGCCGGAACATTAGT<br>GCAGGCAGCTTCCACAGCAATGGCATCCTGGTCATCCAGCGGATAGTTAAT<br>GATCAGCCCACTGACGCGTTGCGCGAGAAGATTGTGCACCGCCGCTTTACA<br>GGCTTCGACGCCGCTTCGTTCTACCATCGACACCACCACGCTGGCACCCAG<br>TTGATCGGCGCGAGATTTAATCGCCGCGACAATTTGCGACGGCGCGTGCAG

	GGCCAGACTGGAGGTGGCAACGCCAATCAGCAACGACTGTTTGCCCGCCA<br>GTTGTTGTGCCACGCGGTTGGGAATGTAATTCAGCTCCGCCATCGCCGCTTC<br>CACTTTTTCCCGCGTTTTCGCAGAAACGTGGCTGGCCTGGTTCACCACGCGG<br>GAAACGGTCTGATAAGAGACACCGGCATACTCTGCGACATCGTATAACGT<br>TACTGGTTTCACATTCACCACCCTGAATTGACTCTCTTCCGGGCGCTATCAT<br>GCCATACCGCGAAAGGTTTTGCGCCATTCGATGGTGTCCGGGATCTCGACG<br>CTCTCCCTTATGAGTGATAGCCGTTTGTCTGGTGTCTACGCCGCGC
Z (pLlacO–STAR Target–3WJ Repressor(RBS)–Linker–GFPmut3b-ASV–T7term–KanR–(Bla Promoter)–ColA origin–backbone)	ATAAATGTGAGCGGATAACATTGACATTGTGAGCGGATAACAAGATACTG<br>AGCACGGAGTTTTTACAGTGAATTGTTTTAATTAGTTGTATAAATGTTGGAG<br>CAGCGGGGAATGTATACAGTTCATGTATATATTCCCCGCTTTTTTTTTGGGA<br>CTAATCAGATCTACTTGTTATAGTTATGAACAGAGGAGACATAACATGAAC<br>AAGCACCTAACAAGACTAATCAACCTGGCGGCAGCGCAAAAGATGCGTA<br>AAGGAGAAGAACTTTTCACTGGAGTTGTCCCAATTCTTGTTGAATTAGATG<br>GTGATGTTAATGGGCACAAATTTTCTGTCAGTGGAGAGGGTGAAGGTGATG<br>CAACATACGGAAAACTTACCCTTAAATTTATTTGCACTACTGGAAAACTAC<br>CTGTTCCGTGGCCAACACTTGTCACTACTTTCGGTTATGGTGTTCAATGCTTT<br>GCGAGATACCCAGATCACATGAAACAGCATGACTTTTTCAAGAGTGCCAT<br>GCCCGAAGGTTACGTACAGGAAAGAACTATATTTTTCAAAGATGACGGGA<br>ACTACAAGACACGTGCTGAAGTCAAGTTTGAAGGTGATACCCTTGTTAATA<br>GAATCGAGTTAAAAGGTATTGATTTTAAAGAAGATGGAAACATTCTTGGAC<br>ACAAATTGGAATACAACTATAACTCACACAATGTATACATCATGGCAGAC<br>AAACAAAAGAATGGAATCAAAGTTAACTTCAAAATTAGACACAACATTGA<br>AGATGGAAGCGTTCAACTAGCAGACCATTATCAACAAAATACTCCGATTG<br>GCGATGGCCCTGTCCTTTTACCAGACAACCATTACCTGTCCACACAATCTG<br>CCCTTTCGAAAGATCCCAACGAAAAGAGAGACCACATGGTCCTTCTTGAGT<br>TTGTAACCGCTGCTGGGATTACACATGGCATGGATGAACTATACAAAAGGC<br>CTGCAGCAAACGACGAAAACTACGCTGCATCAGTTTAATAAGATAAACCA<br>GAGCGGCACGGCAAGCAGAGTATACGAGATTCGGTAGCCACCGCTGAGCA<br>ATAACTAGCATAACCCCTTGGGGCCTCTAAACGGGTCTTGAGGGGTTTTTT<br>GCTGAAACCTCAGGCATTTGAGAAGCACACGGTCACACTGCTTCCGGTAGT<br>CAATAAACCGGTAAACCAGCAATAGACATAAGCGGCTATTTAACGACCCT<br>GCCCTGAACCGACGACAAGCTGACGACCGGGTCTCCGCAAGTGGCACTTTT<br>CGGGGAAATGTGCGCGGAACCCCTATTTGTTTATTTTTCTAAATACATTCAA<br>ATATGTATCCGCTCATGAATTAATTCTTAGAAAAACTCATCGAGCATCAAA<br>TGAAACTGCAATTTATTCATATCAGGATTATCAATACCATATTTTTGAAAAA<br>GCCGTTTCTGTAATGAAGGAGAAAACTCACCGAGGCAGTTCCATAGGATG<br>GCAAGATCCTGGTATCGGTCTGCGATTCCGACTCGTCCAACATCAATACAA<br>CCTATTAATTTCCCCTCGTCAAAAATAAGGTTATCAAGTGAGAAATCACCA<br>TGAGTGACGACTGAATCCGGTGAGAATGGCAAAAGTTTATGCATTTCTTTC<br>CAGACTTGTTCAACAGGCCAGCCATTACGCTCGTCATCAAAATCACTCGCA<br>TCAACCAAACCGTTATTCATTCGTGATTGCGCCTGAGCGAGACGAAATACG<br>CGGTCGCTGTTAAAAGGACAATTACAAACAGGAATCGAATGCAACCGGCG<br>CAGGAACACTGCCAGCGCATCAACAATATTTTCACCTGAATCAGGATATTC<br>TTCTAATACCTGGAATGCTGTTTTCCCGGGGATCGCAGTGGTGAGTAACCA<br>TGCATCATCAGGAGTACGGATAAAATGCTTGATGGTCGGAAGAGGCATAA<br>ATTCCGTCAGCCAGTTTAGTCTGACCATCTCATCTGTAACATCATTGGCAAC<br>GCTACCTTTGCCATGTTTCAGAAACAACTCTGGCGCATCGGGCTTCCCATAC<br>AATCGATAGATTGTCGCACCTGATTGCCCGACATTATCGCGAGCCCATTTA<br>TACCCATATAAATCAGCATCCATGTTGGAATTTAATCGCGGCCTAGAGCAA<br>GACGTTTCCCGTTGAATATGGCTCATACTCTTCCTTTTTCAATATTATTGAAG<br>CATTTATCAGGGTTATTGTCTCATGAGCGGATACATATTTGAATGTATTTAG

	AAAAATAAACAAATAGGCATGCTAGCGCAGAAACGTCCTAGAAGATGCC<br>AGGAGGATACTTAGCAGAGAGACAATAAGGCCGGAGCGAAGCCGTTTTTC<br>CATAGGCTCCGCCCCCCTGACGAACATCACGAAATCTGACGCTCAAATCA<br>GTGGTGGCGAAACCCGACAGGACTATAAAGATACCAGGCGTTTCCCCCTG<br>ATGGCTCCCTCTTGCGCTCTCCTGTTCCCGTCCTGCGGCGTCCGTGTTGTGGT<br>GGAGGCTTTACCCAAATCACCACGTCCCGTTCCGTGTAGACAGTTCGCTCC<br>AAGCTGGGCTGTGTGCAAGAACCCCCCGTTCAGCCCGACTGCTGCGCCTTA<br>TCCGGTAACTATCATCTTGAGTCCAACCCGGAAAGACACGACAAAACGCC<br>ACTGGCAGCAGCCATTGGTAACTGAGAATTAGTGGATTTAGATATCGAGAG<br>TCTTGAAGTGGTGGCCTAACAGAGGCTACACTGAAAGGACAGTATTTGGTA<br>TCTGCGCTCCACTAAAGCCAGTTACCAGGTTAAGCAGTTCCCCAACTGACT<br>TAACCTTCGATCAAACCGCCTCCCCAGGCGGTTTTTTCGTTTACAGAGCAG<br>GAGATTACGACGATCGTAAAAGGATCTCAAGAAGATCCTTTACGGATTCCC<br>GACACCATCACTCTAGATTTCAGTGCAATTTATCTCTTCAAATGTAGCACCT<br>GAAGTCAGCCCCATACGATATAAGTTGTAATTCTCATGTTAGTCATGCCCC<br>GCGCCCACCGGAAGGAGCTGACTGGGTTGAAGGCTCTCAAGGGCATCGGT<br>CGAGATCCCGGTGCCTAATGAGTGAGCTAACTTACATTAATTGCGTTGCGC<br>TCACTGCCCGCTTTCCAGTCGGGAAACCTGTCGTGCCAGCTGCATTAATGA<br>ATCGGCCAACGCGCGGGGAGAGGCGGTTTGCGTATTGGGCGCCAGGGTGG<br>TTTTTCTTTTCACCAGTGAGACGGGCAACAGCTGATTGCCCTTCACCGCCTG<br>GCCCTGAGAGAGTTGCAGCAAGCGGTCCACGCTGGTTTGCCCCAGCAGGC<br>GAAAATCCTGTTTGATGGTGGTTAACGGCGGGATATAACATGAGCTGTCTT<br>CGGTATCGTCGTATCCCACTACCGAGATGTCCGCACCAACGCGCAGCCCGG<br>ACTCGGTAATGGCGCGCATTGCGCCCAGCGCCATCTGATCGTTGGCAACCA<br>GCATCGCAGTGGGAACGATGCCCTCATTCAGCATTTGCATGGTTTGTTGAA<br>AACCGGACATGGCACTCCAGTCGCCTTCCCGTTCCGCTATCGGCTGAATTT<br>GATTGCGAGTGAGATATTTATGCCAGCCAGCCAGACGCAGACGCGCCGAG<br>ACAGAACTTAATGGGCCCGCTAACAGCGCGATTTGCTGGTGACCCAATGC<br>GACCAGATGCTCCACGCCCAGTCGCGTACCGTCTTCATGGGAGAAAATAAT<br>ACTGTTGATGGGTGTCTGGTCAGAGACATCAAGAAATAACGCCGGAACAT<br>TAGTGCAGGCAGCTTCCACAGCAATGGCATCCTGGTCATCCAGCGGATAGT<br>TAATGATCAGCCCACTGACGCGTTGCGCGAGAAGATTGTGCACCGCCGCTT<br>TACAGGCTTCGACGCCGCTTCGTTCTACCATCGACACCACCACGCTGGCAC<br>CCAGTTGATCGGCGCGAGATTTAATCGCCGCGACAATTTGCGACGGCGCGT<br>GCAGGGCCAGACTGGAGGTGGCAACGCCAATCAGCAACGACTGTTTGCCC<br>GCCAGTTGTTGTGCCACGCGGTTGGGAATGTAATTCAGCTCCGCCATCGCC<br>GCTTCCACTTTTTCCCGCGTTTTCGCAGAAACGTGGCTGGCCTGGTTCACCA<br>CGCGGGAAACGGTCTGATAAGAGACACCGGCATACTCTGCGACATCGTAT<br>AACGTTACTGGTTTCACATTCACCACCCTGAATTGACTCTCTTCCGGGCGCT<br>ATCATGCCATACCGCGAAAGGTTTTGCGCCATTCGATGGTGTCCGGGATCT<br>CGACGCTCTCCCTTATGAAGTCTAACGCTGCTCTGGGCTAACTGTC

Supplementary Table S6. Examples of RNA-protein hybrid IFFL DNA plasmid sequences.

Name (architecture)	Sequence
X (pT7–THS Trigger–T7term–(Bla Promoter)–AmpR–pBR322 origin–backbone)	TAATACGACTCACTATAGGGATACACATAGAATCATGTGTATAACACTACT<br>AAACTTCTATCATATTCAATCACTAGCATAACCCCTTGGGGCCTCTAAACG<br>GGTCTTGAGGGGTTTTTTGCTGAAAGGAGGAACTATATCCGGATATCCCGC<br>AAGAGGCCCGGCAGTACCGGCATAACCAAGCCTATGCCTACAGCATCCAG<br>GGTGACGGTGCCGAGGATGACGATGAGCGCATTGTTAGATTTCATACACGG<br>TGCCTGACTGCGTTAGCAATTTAACTGTGATAAACTACCGCATTAAAGCTT<br>ATCGATGATAAGCTGTCAAACATGAGAATTCTTGAAGACGAAAGGGCCTC

	GTGATACGCCTATTTTTATAGGTTAATGTCATGATAATAATGGTTTCTTAGA<br>CGTCAGGTGGCACTTTTCGGGGAAATGTGCGCGGAACCCCTATTTGTTTATT<br>TTTCTAAATACATTCAAATATGTATCCGCTCATGAGACAATAACCCTGATA<br>AATGCTTCAATAATATTGAAAAAGGAAGAGTATGAGTATTCAACATTTCCG<br>TGTCGCCCTTATTCCCTTTTTTGCGGCATTTTGCCTTCCTGTTTTTGCTCACCC<br>AGAAACGCTGGTGAAAGTAAAAGATGCTGAAGATCAGTTGGGTGCACGAG<br>TGGGTTACATCGAACTGGATCTCAACAGCGGTAAGATCCTTGAGAGTTTTC<br>GCCCCGAAGAACGTTTTCCAATGATGAGCACTTTTAAAGTTCTGCTATGTG<br>GCGCGGTATTATCCCGTGTTGACGCCGGGCAAGAGCAACTCGGTCGCCGC<br>ATACACTATTCTCAGAATGACTTGGTTGAGTACTCACCAGTCACAGAAAAG<br>CATCTTACGGATGGCATGACAGTAAGAGAATTATGCAGTGCTGCCATAACC<br>ATGAGTGATAACACTGCGGCCAACTTACTTCTGACAACGATCGGAGGACC<br>GAAGGAGCTAACCGCTTTTTTGCACAACATGGGGGATCATGTAACTCGCCT<br>TGATCGTTGGGAACCGGAGCTGAATGAAGCCATACCAAACGACGAGCGTG<br>ACACCACGATGCCTGCAGCAATGGCAACAACGTTGCGCAAACTATTAACT<br>GGCGAACTACTTACTCTAGCTTCCCGGCAACAATTAATAGACTGGATGGAG<br>GCGGATAAAGTTGCAGGACCACTTCTGCGCTCGGCCCTTCCGGCTGGCTGG<br>TTTATTGCTGATAAATCTGGAGCCGGTGAGCGTGGGTCTCGCGGTATCATTG<br>CAGCACTGGGGCCAGATGGTAAGCCCTCCCGTATCGTAGTTATCTACACGA<br>CGGGGAGTCAGGCAACTATGGATGAACGAAATAGACAGATCGCTGAGATA<br>GGTGCCTCACTGATTAAGCATTGGTAACTGTCAGACCAAGTTTACTCATAT<br>ATACTTTAGATTGATTTAAAACTTCATTTTTAATTTAAAAGGATCTAGGTGA<br>AGATCCTTTTTGATAATCTCATGACCAAAATCCCTTAACGTGAGTTTTCGTT<br>CCACTGAGCGTCAGACCCCGTAGAAAAGATCAAAGGATCTTCTTGAGATC<br>CTTTTTTTCTGCGCGTAATCTGCTGCTTGCAAACAAAAAAACCACCGCTACC<br>AGCGGTGGTTTGTTTGCCGGATCAAGAGCTACCAACTCTTTTTCCGAAGGT<br>AACTGGCTTCAGCAGAGCGCAGATACCAAATACTGTCCTTCTAGTGTAGCC<br>GTAGTTAGGCCACCACTTCAAGAACTCTGTAGCACCGCCTACATACCTCGC<br>TCTGCTAATCCTGTTACCAGTGGCTGCTGCCAGTGGCGATAAGTCGTGTCTT<br>ACCGGGTTGGACTCAAGACGATAGTTACCGGATAAGGCGCAGCGGTCGGG<br>CTGAACGGGGGGTTCGTGCACACAGCCCAGCTTGGAGCGAACGACCTACA<br>CCGAACTGAGATACCTACAGCGTGAGCTATGAGAAAGCGCCACGCTTCCC<br>GAAGGGAGAAAGGCGGACAGGTATCCGGTAAGCGGCAGGGTCGGAACAG<br>GAGAGCGCACGAGGGAGCTTCCAGGGGGAAACGCCTGGTATCTTTATAGT<br>CCTGTCGGGTTTCGCCACCTCTGACTTGAGCGTCGATTTTTGTGATGCTCGT<br>CAGGGGGGCGGAGCCTATGGAAAAACGCCAGCAACGCGGCCTTTTTACGG<br>TTCCTGGCCTTTTGCTGGCCTTTTGCTCACATGTTCTTTCCTGCGTTATCCCCT<br>GATTCTGTGGATAACCGTATTACCGCCTTTGAGTGAGCTGATACCGCTCGCC<br>GCAGCCGAACGACCGAGCGCAGCGAGTCAGTGAGCGAGGAAGCGGAAGA<br>GCGCCTGATGCGGTATTTTCTCCTTACGCATCTGTGCGGTATTTCACACCGC<br>ATATATGGTGCACTCTCAGTACAATCTGCTCTGATGCCGCATAGTTAAGCC<br>AGTATACACTCCGCTATCGCTACGTGACTGGGTCATGGCTGCGCCCCGACA<br>CCCGCCAACACCCGCTGACGCGCCCTGACGGGCTTGTCTGCTCCCGGCATC<br>CGCTTACAGACAAGCTGTGACCGTCTCCGGGAGCTGCATGTGTCAGAGGTT<br>TTCACCGTCATCACCGAAACGCGCGAGGCAGCTGCGGTAAAGCTCATCAG<br>CGTGGTCGTGAAGCGATTCACAGATGTCTGCCTGTTCATCCGCGTCCAGCT<br>CGTTGAGTTTCTCCAGAAGCGTTAATGTCTGGCTTCTGATAAAGCGGGCCA<br>TGTTAAGGGCGGTTTTTTCCTGTTTGGTCACTGATGCCTCCGTGTAAGGGGG<br>ATTTCTGTTCATGGGGGTAATGATACCGATGAAACGAGAGAGGATGCTCAC<br>GATACGGGTTACTGATGATGAACATGCCCGGTTACTGGAACGTTGTGAGGG<br>TAAACAACTGGCGGTATGGATGCGGCGGGACCAGAGAAAAATCACTCAGG

	GTCAATGCCAGCGCTTCGTTAATACAGATGTAGGTGTTCCACAGGGTAGCC<br>AGCAGCATCCTGCGATGCAGATCCGGAACATAATGGTGCAGGGCGCTGAC<br>TTCCGCGTTTCCAGACTTTACGAAACACGGAAACCGAAGACCATTCATGTT<br>GTTGCTCAGGTCGCAGACGTTTTGCAGCAGCAGTCGCTTCACGTTCGCTCGC<br>GTATCGGTGATTCATTCTGCTAACCAGTAAGGCAACCCCGCCAGCCTAGCC<br>GGGTCCTCAACGACAGGAGCACGATCATGCGCACCCGTGGCCAGGACCCA<br>ACGCTGCCCGAGATGCGCCGCGTGCGGCTGCTGGAGATGGCGGACGCGAT<br>GGATATGTTCTGCCAAGGGTTGGTTTGCGCATTCACAGTTCTCCGCAAGAA<br>TTGATTGGCTCCAATTCTTGGAGTGGTGAATCCGTTAGCGAGGTGCCGCCG<br>GCTTCCATTCAGGTCGAGGTGGCCCGGCTCCATGCACCGCGACGCAACGCG<br>GGGAGGCAGACAAGGTATAGGGCGGCGCCTACAATCCATGCCAACCCGTT<br>CCATGTGCTCGCCGAGGCGGCATAAATCGCCGTGACGATCAGCGGTCCAG<br>TGATCGAAGTTAGGCTGGTAAGAGCCGCGAGCGATCCTTGAAGCTGTCCCT<br>GATGGTCGTCATCTACCTGCCTGGACAGCATGGCCTGCAACGCGGGCATCC<br>CGATGCCGCCGGAAGCGAGAAGAATCATAATGGGGAAGGCCATCCAGCCT<br>CGCGTCGCGAACGCCAGCAAGACGTAGCCCAGCGCGTCGGCCGCCATGCC<br>GGCGATAATGGCCTGCTTCTCGCCGAAACGTTTGGTGGCGGGACCAGTGAC<br>GAAGGCTTGAGCGAGGGCGTGCAAGATTCCGAATACCGCAAGCGACAGGC<br>CGATCATCGTCGCGCTCCAGCGAAAGCGGTCCTCGCCGAAAATGACCCAG<br>AGCGCTGCCGGCACCTGTCCTACGAGTTGCATGATAAAGAAGACAGTCAT<br>AAGTGCGGCGACGATAGTCATGCCCCGCGCCCACCGGAAGGAGCTGACTG<br>GGTTGAAGGCTCTCAAGGGCATCGGTCGAGATCCCGGTGCCTAATGAGTG<br>AGCTAACTTACATTAATTGCGTTGCGCTCACTGCCCGCTTTCCAGTCGGGAA<br>ACCTGTCGTGCCAGCTGCATTAATGAATCGGCCAACGCGCGGGGAGAGGC<br>GGTTTGCGTATTGGGCGCCAGGGTGGTTTTTCTTTTCACCAGTGAGACGGGC<br>AACAGCTGATTGCCCTTCACCGCCTGGCCCTGAGAGAGTTGCAGCAAGCG<br>GTCCACGCTGGTTTGCCCCAGCAGGCGAAAATCCTGTTTGATGGTGGTTAA<br>CGGCGGGATATAACATGAGCTGTCTTCGGTATCGTCGTATCCCACTACCGA<br>GATATCCGCACCAACGCGCAGCCCGGACTCGGTAATGGCGCGCATTGCGC<br>CCAGCGCCATCTGATCGTTGGCAACCAGCATCGCAGTGGGAACGATGCCCT<br>CATTCAGCATTTGCATGGTTTGTTGAAAACCGGACATGGCACTCCAGTCGC<br>CTTCCCGTTCCGCTATCGGCTGAATTTGATTGCGAGTGAGATATTTATGCCA<br>GCCAGCCAGACGCAGACGCGCCGAGACAGAACTTAATGGGCCCGCTAAC<br>AGCGCGATTTGCTGGTGACCCAATGCGACCAGATGCTCCACGCCCAGTCGC<br>GTACCGTCTTCATGGGAGAAAATAATACTGTTGATGGGTGTCTGGTCAGAG<br>ACATCAAGAAATAACGCCGGAACATTAGTGCAGGCAGCTTCCACAGCAAT<br>GGCATCCTGGTCATCCAGCGGATAGTTAATGATCAGCCCACTGACGCGTTG<br>CGCGAGAAGATTGTGCACCGCCGCTTTACAGGCTTCGACGCCGCTTCGTTC<br>TACCATCGACACCACCACGCTGGCACCCAGTTGATCGGCGCGAGATTTAAT<br>CGCCGCGACAATTTGCGACGGCGCGTGCAGGGCCAGACTGGAGGTGGCAA<br>CGCCAATCAGCAACGACTGTTTGCCCGCCAGTTGTTGTGCCACGCGGTTGG<br>GAATGTAATTCAGCTCCGCCATCGCCGCTTCCACTTTTTCCCGCGTTTTCGC<br>AGAAACGTGGCTGGCCTGGTTCACCACGCGGGAAACGGTCTGATAAGAGA<br>CACCGGCATACTCTGCGACATCGTATAACGTTACTGGTTTCACATTCACCA<br>CCCTGAATTGACTCTCTTCCGGGCGCTATCATGCCATACCGCGAAAGGTTTT<br>GCGCCATTCGATGGTGTCCGGGATCTCGACGCTCTCCCTTATGAACGTGTA<br>CGGGCTATCTGGCTTTCGTTGCGC
Y_ASV (pJ23116–THS(RBS)–Linker–TetR–ASV–T7term–SpecR–(Bla Promoter)–CloDF13 origin–backbone)	TTGACAGCTAGCTCAGTCCTAGGGACTATGCTAGCGATTGAATATGATAGA<br>AGTTTAGTAGTAGACAATAGAACAGAGGAGATATTGATGACTACTAAACT<br>AAACCTGGCGGCAGCGCAAAAGATGTCTAGATTAGATAAAAGTAAAGTGA<br>TTAACAGCGCATTAGAGCTGCTTAATGAGGTCGGAATCGAAGGTTTAACAA

	CCCGTAAACTCGCCCAGAAGCTAGGTGTAGAGCAGCCTACATTGTATTGGC<br>ATGTAAAAAATAAGCGGGCTTTGCTCGACGCCTTAGCCATTGAGATGTTAG<br>ATAGGCACCATACTCACTTTTGCCCTTTAGAAGGGGAAAGCTGGCAAGATT<br>TTTTACGTAATAACGCTAAAAGTTTTAGATGTGCTTTACTAAGTCATCGCGA<br>TGGAGCAAAAGTACATTTAGGTACACGGCCTACAGAAAAACAGTATGAAA<br>CTCTCGAAAATCAATTAGCCTTTTTATGCCAACAAGGTTTTTCACTAGAGAA<br>TGCATTATATGCACTCAGCGCTGTGGGGCATTTTACTTTAGGTTGCGTATTG<br>GAAGATCAAGAGCATCAAGTCGCTAAAGAAGAAAGGGAAACACCTACTA<br>CTGATAGTATGCCGCCATTATTACGACAAGCTATCGAATTATTTGATCACC<br>AAGGTGCAGAGCCAGCCTTCTTATTCGGCCTTGAATTGATCATATGCGGAT<br>TAGAAAAACAACTTAAATGTGAAAGTGGGTCTAGGCCTGCAGCAAACGAC<br>GAAAACTACGCTGCATCAGTTTAATAAGATAAACCAGAGCGGCACGGCAA<br>GCAGAGTATACGAGATTCGGTAGCCACCGCTGAGCAATAACTAGCATAAC<br>CCCTTGGGGCCTCTAAACGGGTCTTGAGGGGTTTTTTGCTGAAACCTCAGG<br>CATTTGAGAAGCACACGGTCACACTGCTTCCGGTAGTCAATAAACCGGTAA<br>ACCAGCAATAGACATAAGCGGCTATTTAACGACCCTGCCCTGAACCGACG<br>ACCGGGTCATCGTGGCCGGATCTTGCGGCCCCTCGGCTTGAACGAATTGTT<br>AGACATTATTTGCCGACTACCTTGGTGATCTCGCCTTTCACGTAGTGGACAA<br>ATTCTTCCAACTGATCTGCGCGCGAGGCCAAGCGATCTTCTTCTTGTCCAAG<br>ATAAGCCTGTCTAGCTTCAAGTATGACGGGCTGATACTGGGCCGGCAGGCG<br>CTCCATTGCCCAGTCGGCAGCGACATCCTTCGGCGCGATTTTGCCGGTTACT<br>GCGCTGTACCAAATGCGGGACAACGTAAGCACTACATTTCGCTCATCGCCA<br>GCCCAGTCGGGCGGCGAGTTCCATAGCGTTAAGGTTTCATTTAGCGCCTCA<br>AATAGATCCTGTTCAGGAACCGGATCAAAGAGTTCCTCCGCCGCTGGACCT<br>ACCAAGGCAACGCTATGTTCTCTTGCTTTTGTCAGCAAGATAGCCAGATCA<br>ATGTCGATCGTGGCTGGCTCGAAGATACCTGCAAGAATGTCATTGCGCTGC<br>CATTCTCCAAATTGCAGTTCGCGCTTAGCTGGATAACGCCACGGAATGATG<br>TCGTCGTGCACAACAATGGTGACTTCTACAGCGCGGAGAATCTCGCTCTCT<br>CCAGGGGAAGCCGAAGTTTCCAAAAGGTCGTTGATCAAAGCTCGCCGCGT<br>TGTTTCATCAAGCCTTACGGTCACCGTAACCAGCAAATCAATATCACTGTG<br>TGGCTTCAGGCCGCCATCCACTGCGGAGCCGTACAAATGTACGGCCAGCA<br>ACGTCGGTTCGAGATGGCGCTCGATGACGCCAACTACCTCTGATAGTTGAG<br>TCGATACTTCGGCGATCACCGCTTCCCTCATACTCTTCCTTTTTCAATATTAT<br>TGAAGCATTTATCAGGGTTATTGTCTCATGAGCGGATACATATTTGAATGTA<br>TTTAGAAAAATAAACAAATAGCTAGCTCACTCGGTCGCTACGCTCCGGGCG<br>TGAGACTGCGGCGGGCGCTGCGGACACATACAAAGTTACCCACAGATTCC<br>GTGGATAAGCAGGGGACTAACATGTGAGGCAAAACAGCAGGGCCGCGCC<br>GGTGGCGTTTTTCCATAGGCTCCGCCCTCCTGCCAGAGTTCACATAAACAG<br>ACGCTTTTCCGGTGCATCTGTGGGAGCCGTGAGGCTCAACCATGAATCTGA<br>CAGTACGGGCGAAACCCGACAGGACTTAAAGATCCCCACCGTTTCCGGCG<br>GGTCGCTCCCTCTTGCGCTCTCCTGTTCCGACCCTGCCGTTTACCGGATACC<br>TGTTCCGCCTTTCTCCCTTACGGGAAGTGTGGCGCTTTCTCATAGCTCACAC<br>ACTGGTATCTCGGCTCGGTGTAGGTCGTTCGCTCCAAGCTGGGCTGTAAGC<br>AAGAACTCCCCGTTCAGCCCGACTGCTGCGCCTTATCCGGTAACTGTTCAC<br>TTGAGTCCAACCCGGAAAAGCACGGTAAAACGCCACTGGCAGCAGCCATT<br>GGTAACTGGGAGTTCGCAGAGGATTTGTTTAGCTAAACACGCGGTTGCTCT<br>TGAAGTGTGCGCCAAAGTCCGGCTACACTGGAAGGACAGATTTGGTTGCTG<br>TGCTCTGCGAAAGCCAGTTACCACGGTTAAGCAGTTCCCCAACTGACTTAA<br>CCTTCGATCAAACCACCTCCCCAGGTGGTTTTTTCGTTTACAGGGCAAAAG<br>ATTACGCGCAGAAAAAAAGGATCTCAAGAAGATCCTTTGATCTTTTCTACT<br>GAACCGCTCTAGATTTCAGTGCAATTTATCTCTTCAAATGTAGCACCTGAA

	GTCAGCCCCATACGATATAAGTTGTAATTCTCATGTTAGTCATGCCCCGCGC<br>CCACCGGAAGGAGCTGACTGGGTTGAAGGCTCTCAAGGGCATCGGTCGAG<br>ATCCCGGTGCCTAATGAGTGAGCTAACTTACATTAATTGCGTTGCGCTCACT<br>GCCCGCTTTCCAGTCGGGAAACCTGTCGTGCCAGCTGCATTAATGAATCGG<br>CCAACGCGCGGGGAGAGGCGGTTTGCGTATTGGGCGCCAGGGTGGTTTTTC<br>TTTTCACCAGTGAGACGGGCAACAGCTGATTGCCCTTCACCGCCTGGCCCT<br>GAGAGAGTTGCAGCAAGCGGTCCACGCTGGTTTGCCCCAGCAGGCGAAAA<br>TCCTGTTTGATGGTGGTTAACGGCGGGATATAACATGAGCTGTCTTCGGTAT<br>CGTCGTATCCCACTACCGAGATGTCCGCACCAACGCGCAGCCCGGACTCG<br>GTAATGGCGCGCATTGCGCCCAGCGCCATCTGATCGTTGGCAACCAGCATC<br>GCAGTGGGAACGATGCCCTCATTCAGCATTTGCATGGTTTGTTGAAAACCG<br>GACATGGCACTCCAGTCGCCTTCCCGTTCCGCTATCGGCTGAATTTGATTGC<br>GAGTGAGATATTTATGCCAGCCAGCCAGACGCAGACGCGCCGAGACAGAA<br>CTTAATGGGCCCGCTAACAGCGCGATTTGCTGGTGACCCAATGCGACCAGA<br>TGCTCCACGCCCAGTCGCGTACCGTCTTCATGGGAGAAAATAATACTGTTG<br>ATGGGTGTCTGGTCAGAGACATCAAGAAATAACGCCGGAACATTAGTGCA<br>GGCAGCTTCCACAGCAATGGCATCCTGGTCATCCAGCGGATAGTTAATGAT<br>CAGCCCACTGACGCGTTGCGCGAGAAGATTGTGCACCGCCGCTTTACAGGC<br>TTCGACGCCGCTTCGTTCTACCATCGACACCACCACGCTGGCACCCAGTTG<br>ATCGGCGCGAGATTTAATCGCCGCGACAATTTGCGACGGCGCGTGCAGGG<br>CCAGACTGGAGGTGGCAACGCCAATCAGCAACGACTGTTTGCCCGCCAGT<br>TGTTGTGCCACGCGGTTGGGAATGTAATTCAGCTCCGCCATCGCCGCTTCC<br>ACTTTTTCCCGCGTTTTCGCAGAAACGTGGCTGGCCTGGTTCACCACGCGGG<br>AAACGGTCTGATAAGAGACACCGGCATACTCTGCGACATCGTATAACGTT<br>ACTGGTTTCACATTCACCACCCTGAATTGACTCTCTTCCGGGCGCTATCATG<br>CCATACCGCGAAAGGTTTTGCGCCATTCGATGGTGTCCGGGATCTCGACGC<br>TCTCCCTTATGAGTGATAGCCGTTTGTCTGGTGTCTACGCCGCGCGGCTAA<br>CTGTC
Z (pT7–TetO–THS(RBS)–Linker–GFPmut3b-ASV–T7term–KanR–(Bla Promoter)–ColA origin–backbone)	TAATACGACTCACTATAGGTCTATCATTGATAGGGTTTGATTGAATATGATA<br>GAAGTTTAGTAGTAGACAATAGAACAGAGGAGATATTGATGACTACTAAA<br>CTAAACCTGGCGGCAGCGCAAAAGATGCGTAAAGGAGAAGAACTTTTCAC<br>TGGAGTTGTCCCAATTCTTGTTGAATTAGATGGTGATGTTAATGGGCACAA<br>ATTTTCTGTCAGTGGAGAGGGTGAAGGTGATGCAACATACGGAAAACTTAC<br>CCTTAAATTTATTTGCACTACTGGAAAACTACCTGTTCCGTGGCCAACACTT<br>GTCACTACTTTCGGTTATGGTGTTCAATGCTTTGCGAGATACCCAGATCACA<br>TGAAACAGCATGACTTTTTCAAGAGTGCCATGCCCGAAGGTTACGTACAGG<br>AAAGAACTATATTTTTCAAAGATGACGGGAACTACAAGACACGTGCTGAA<br>GTCAAGTTTGAAGGTGATACCCTTGTTAATAGAATCGAGTTAAAAGGTATT<br>GATTTTAAAGAAGATGGAAACATTCTTGGACACAAATTGGAATACAACTAT<br>AACTCACACAATGTATACATCATGGCAGACAAACAAAAGAATGGAATCAA<br>AGTTAACTTCAAAATTAGACACAACATTGAAGATGGAAGCGTTCAACTAG<br>CAGACCATTATCAACAAAATACTCCGATTGGCGATGGCCCTGTCCTTTTAC<br>CAGACAACCATTACCTGTCCACACAATCTGCCCTTTCGAAAGATCCCAACG<br>AAAAGAGAGACCACATGGTCCTTCTTGAGTTTGTAACCGCTGCTGGGATTA<br>CACATGGCATGGATGAACTATACAAAAGGCCTGCAGCAAACGACGAAAA<br>CTACGCTGCATCAGTTTAATAAGATAAACCAGAGCGGCACGGCAAGCAGA<br>GTATACGAGATTCGGTAGCCACCGCTGAGCAATAACTAGCATAACCCCTTG<br>GGGCCTCTAAACGGGTCTTGAGGGGTTTTTTGCTGAAACCTCAGGCATTTG<br>AGAAGCACACGGTCACACTGCTTCCGGTAGTCAATAAACCGGTAAACCAG<br>CAATAGACATAAGCGGCTATTTAACGACCCTGCCCTGAACCGACGACAAG<br>CTGACGACCGGGTCTCCGCAAGTGGCACTTTTCGGGGAAATGTGCGCGGA

ACCCCTATTTGTTTATTTTTCTAAATACATTCAAATATGTATCCGCTCATGAA
TTAATTCTTAGAAAAACTCATCGAGCATCAAATGAAACTGCAATTTATTCA
TATCAGGATTATCAATACCATATTTTTGAAAAAGCCGTTTCTGTAATGAAG
GAGAAAACTCACCGAGGCAGTTCCATAGGATGGCAAGATCCTGGTATCGG
TCTGCGATTCCGACTCGTCCAACATCAATACAACCTATTAATTTCCCCTCGT
CAAAAATAAGGTTATCAAGTGAGAAATCACCATGAGTGACGACTGAATCC
GGTGAGAATGGCAAAAGTTTATGCATTTCTTTCCAGACTTGTTCAACAGGC
CAGCCATTACGCTCGTCATCAAAATCACTCGCATCAACCAAACCGTTATTC
ATTCGTGATTGCGCCTGAGCGAGACGAAATACGCGGTCGCTGTTAAAAGG
ACAATTACAAACAGGAATCGAATGCAACCGGCGCAGGAACACTGCCAGC
GCATCAACAATATTTTCACCTGAATCAGGATATTCTTCTAATACCTGGAATG
CTGTTTTCCCGGGGATCGCAGTGGTGAGTAACCATGCATCATCAGGAGTAC
GGATAAAATGCTTGATGGTCGGAAGAGGCATAAATTCCGTCAGCCAGTTTA
GTCTGACCATCTCATCTGTAACATCATTGGCAACGCTACCTTTGCCATGTTT
CAGAAACAACTCTGGCGCATCGGGCTTCCCATACAATCGATAGATTGTCGC
ACCTGATTGCCCGACATTATCGCGAGCCCATTTATACCCATATAAATCAGC
ATCCATGTTGGAATTTAATCGCGGCCTAGAGCAAGACGTTTCCCGTTGAAT
ATGGCTCATACTCTTCCTTTTTCAATATTATTGAAGCATTTATCAGGGTTATT
GTCTCATGAGCGGATACATATTTGAATGTATTTAGAAAAATAAACAAATAG
GCATGCTAGCGCAGAAACGTCCTAGAAGATGCCAGGAGGATACTTAGCAG
AGAGACAATAAGGCCGGAGCGAAGCCGTTTTTCCATAGGCTCCGCCCCCCT
GACGAACATCACGAAATCTGACGCTCAAATCAGTGGTGGCGAAACCCGAC
AGGACTATAAAGATACCAGGCGTTTCCCCCTGATGGCTCCCTCTTGCGCTCT
CCTGTTCCCGTCCTGCGGCGTCCGTGTTGTGGTGGAGGCTTTACCCAAATCA
CCACGTCCCGTTCCGTGTAGACAGTTCGCTCCAAGCTGGGCTGTGTGCAAG
AACCCCCCGTTCAGCCCGACTGCTGCGCCTTATCCGGTAACTATCATCTTGA
GTCCAACCCGGAAAGACACGACAAAACGCCACTGGCAGCAGCCATTGGTA
ACTGAGAATTAGTGGATTTAGATATCGAGAGTCTTGAAGTGGTGGCCTAAC
AGAGGCTACACTGAAAGGACAGTATTTGGTATCTGCGCTCCACTAAAGCC
AGTTACCAGGTTAAGCAGTTCCCCAACTGACTTAACCTTCGATCAAACCGC
CTCCCCAGGCGGTTTTTTCGTTTACAGAGCAGGAGATTACGACGATCGTAA
AAGGATCTCAAGAAGATCCTTTACGGATTCCCGACACCATCACTCTAGATT
TCAGTGCAATTTATCTCTTCAAATGTAGCACCTGAAGTCAGCCCCATACGA
TATAAGTTGTAATTCTCATGTTAGTCATGCCCCGCGCCCACCGGAAGGAGC
TGACTGGGTTGAAGGCTCTCAAGGGCATCGGTCGAGATCCCGGTGCCTAAT
GAGTGAGCTAACTTACATTAATTGCGTTGCGCTCACTGCCCGCTTTCCAGTC
GGGAAACCTGTCGTGCCAGCTGCATTAATGAATCGGCCAACGCGCGGGGA
GAGGCGGTTTGCGTATTGGGCGCCAGGGTGGTTTTTCTTTTCACCAGTGAGA
CGGGCAACAGCTGATTGCCCTTCACCGCCTGGCCCTGAGAGAGTTGCAGCA
AGCGGTCCACGCTGGTTTGCCCCAGCAGGCGAAAATCCTGTTTGATGGTGG
TTAACGGCGGGATATAACATGAGCTGTCTTCGGTATCGTCGTATCCCACTA
CCGAGATGTCCGCACCAACGCGCAGCCCGGACTCGGTAATGGCGCGCATT
GCGCCCAGCGCCATCTGATCGTTGGCAACCAGCATCGCAGTGGGAACGAT
GCCCTCATTCAGCATTTGCATGGTTTGTTGAAAACCGGACATGGCACTCCA
GTCGCCTTCCCGTTCCGCTATCGGCTGAATTTGATTGCGAGTGAGATATTTA
TGCCAGCCAGCCAGACGCAGACGCGCCGAGACAGAACTTAATGGGCCCGC
TAACAGCGCGATTTGCTGGTGACCCAATGCGACCAGATGCTCCACGCCCAG
TCGCGTACCGTCTTCATGGGAGAAAATAATACTGTTGATGGGTGTCTGGTC
AGAGACATCAAGAAATAACGCCGGAACATTAGTGCAGGCAGCTTCCACAG
CAATGGCATCCTGGTCATCCAGCGGATAGTTAATGATCAGCCCACTGACGC
GTTGCGCGAGAAGATTGTGCACCGCCGCTTTACAGGCTTCGACGCCGCTTC

	GTTCTACCATCGACACCACCACGCTGGCACCCAGTTGATCGGCGCGAGATT TAATCGCCGCGACAATTTGCGACGGCGCGTGCAGGGCCAGACTGGAGGTG GCAACGCCAATCAGCAACGACTGTTTGCCCGCCAGTTGTTGTGCCACGCGG TTGGGAATGTAATTCAGCTCCGCCATCGCCGCTTCCACTTTTTCCCGCGTTTT CGCAGAAACGTGGCTGGCCTGGTTCACCACGCGGGAAACGGTCTGATAAG AGACACCGGCATACTCTGCGACATCGTATAACGTTACTGGTTTCACATTCA CCACCCTGAATTGACTCTCTTCCGGGCGCTATCATGCCATACCGCGAAAGG TTTTGCGCCATTCGATGGTGTCCGGGATCTCGACGCTCTCCCTTATGAAGTC TAACGCTGCTCTGGGCTAACTGTCGCGC

Supplementary Table S7. Promoter sequences used in this study. Plasmid sequences can be constructed by replacing the yellow region in the example plasmids in Table S5 and S6 with the yellow region indicated here.

Name	Sequence
pT7	TAATACGACTCACTATAGG
pJ23116	TTGACAGCTAGCTCAGTCCTAGGGACTATGCTAGC
pLlacO	ATAAATGTGAGCGGATAACATTGACATTGTGAGCGG ATAACAAGATACTGAGCACGG

Supplementary Table S8. Insert sequences used in this study. Plasmid sequences can be constructed by replacing the grey region in the example plasmids in Table S5 and S6 with the grey region indicated here (excluding Z).

Name	Sequence
STAR Target–RiboJ–3WJ Trigger	AGTTTTTACAGTGAATTGTTTTAATTAGTTGTATAAATG TTGGAGCAGCGGGGAATGTATACAGTTCATGTATATAT TCCCCGCTTTTTTTTTACGGGAATTAGAAGCTGTCACCG GATGTGCTTTCCGGTCTGATGAGTCCGTGAGGACGAAA CAGCCTCTACAAATAATTTGTTTAAAAACATAACGAA GGGACCTAACATAAACTTGTTAGGTGCGTAGATCTGAT TAGTGTG
Decoy	TCTCACGCCCTCAGCTGGGCGTGAGATGAGCCTCGTCT CCAGATGACGAGGCAACGTAGGATCTGACTGATCCTAC TAT
AAV	GCAGCAAACGACGAAAACTACGCTGCAGCAGTT
LVA	GCAGCAAACGACGAAAACTACGCTCTAGTTGCA

Supplementary Table S9. Other accessary sequences used in this study. Accessary sequences used for constructing plasmids are indicated here.

Name	Sequence
RBS	AGAGGAGA
Linker	AACCTGGCGGCAGCGCAAAAG
T7term	TAGCATAACCCCTTGGGGCCTCTAAACGGGTCTTGAGG GGTTTTTTG
GFPmut3b-ASV	ATGCGTAAAGGAGAAGAACTTTTCACTGGAGTTGTCCC AATTCTTGTTGAATTAGATGGTGATGTTAATGGGCACA AATTTTCTGTCAGTGGAGAGGGTGAAGGTGATGCAACA TACGGAAAACTTACCCTTAAATTTATTTGCACTACTGG AAAACTACCTGTTCCGTGGCCAACACTTGTCACTACTT TCGGTTATGGTGTTCAATGCTTTGCGAGATACCCAGAT CACATGAAACAGCATGACTTTTTCAAGAGTGCCATGCC CGAAGGTTACGTACAGGAAAGAACTATATTTTTCAAAG ATGACGGGAACTACAAGACACGTGCTGAAGTCAAGTT TGAAGGTGATACCCTTGTTAATAGAATCGAGTTAAAAG GTATTGATTTTAAAGAAGATGGAAACATTCTTGGACAC

	AAATTGGAATACAACTATAACTCACACAATGTATACAT<br>CATGGCAGACAAACAAAAGAATGGAATCAAAGTTAAC<br>TTCAAAATTAGACACAACATTGAAGATGGAAGCGTTCA<br>ACTAGCAGACCATTATCAACAAAATACTCCGATTGGCG<br>ATGGCCCTGTCCTTTTACCAGACAACCATTACCTGTCC<br>ACACAATCTGCCCTTTCGAAAGATCCCAACGAAAAGA<br>GAGACCACATGGTCCTTCTTGAGTTTGTAACCGCTGCT<br>GGGATTACACATGGCATGGATGAACTATACAAAAGGC<br>CTGCAGCAAACGACGAAAACTACGCTGCATCAGTTTAA<br>TAA
TetO	TCTATCATTGATAGGGTTT
TetR	ATGTCTAGATTAGATAAAAGTAAAGTGATTAACAGCGC<br>ATTAGAGCTGCTTAATGAGGTCGGAATCGAAGGTTTAA<br>CAACCCGTAAACTCGCCCAGAAGCTAGGTGTAGAGCA<br>GCCTACATTGTATTGGCATGTAAAAAATAAGCGGGCTT<br>TGCTCGACGCCTTAGCCATTGAGATGTTAGATAGGCAC<br>CATACTCACTTTTGCCCTTTAGAAGGGGAAAGCTGGCA<br>AGATTTTTTACGTAATAACGCTAAAAGTTTTAGATGTG<br>CTTTACTAAGTCATCGCGATGGAGCAAAAGTACATTTA<br>GGTACACGGCCTACAGAAAAACAGTATGAAACTCTCG<br>AAAATCAATTAGCCTTTTTATGCCAACAAGGTTTTTCAC<br>TAGAGAATGCATTATATGCACTCAGCGCTGTGGGGCAT<br>TTTACTTTAGGTTGCGTATTGGAAGATCAAGAGCATCA<br>AGTCGCTAAAGAAGAAAGGGAAACACCTACTACTGAT<br>AGTATGCCGCCATTATTACGACAAGCTATCGAATTATT<br>TGATCACCAAGGTGCAGAGCCAGCCTTCTTATTCGGCC<br>TTGAATTGATCATATGCGGATTAGAAAAACAACTTAAA<br>TGTGAAAGTGGGTCT

Supplementary Figures

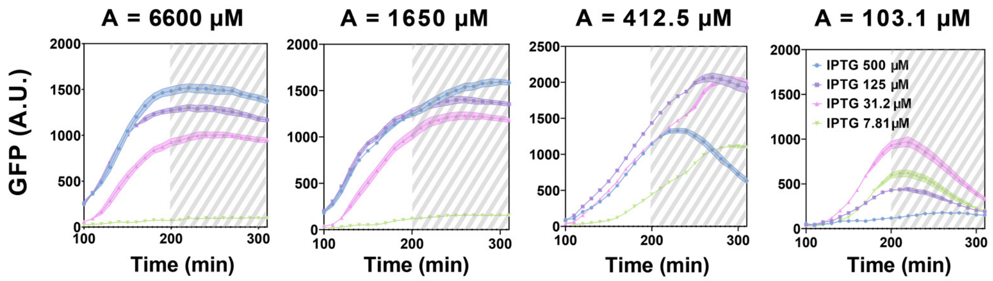

Supplementary Figure S1. *In vivo* characterization of the X to Z activation in the RNA-only IFFL circuit. Time course of GFP fluorescence measurements for different inducer concentrations; IPTG concentrations at 500 μM, 125 μM, 31.2 μM and 7.81 μM; Arabinose concentrations at 6600 μM, 1650 μM, 412.5 μM and 103.125 μM. Data for the first 90 mins were removed due to low OD600 values, and and the time points beyond 200 mins are marked as gray dashed area to indicate the transition to stationary phase. Graphs were represented by different symbols according to IPTG concentrations; 500 μM by blue circles, 125 μM by purple squares, 31.2 μM by pink triangles and 7.81 μM Constitutive by green triangles. The letter 'A' represents Arabinose. Relative errors for GFP fluorescence are from the standard deviation of three biological replicates.

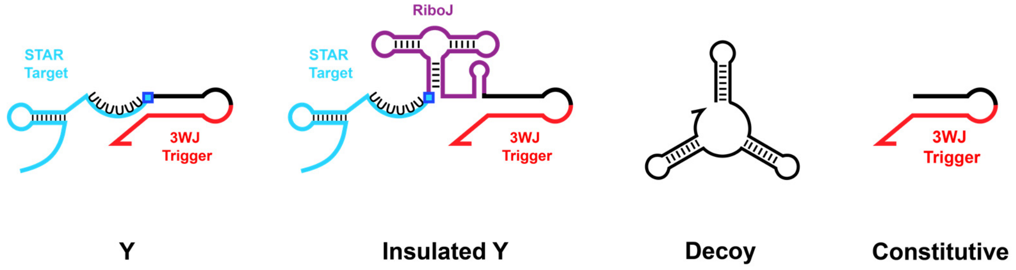

Supplementary Figure S2. RNA secondary structures for four different variants of Y in the RNA-only IFFL circuit. STAR is directly connected to the 3WJ trigger in Regular Y. RiboJ is located between the STAR target and the 3WJ trigger in insulated Y, and when transcribed, the STAR target is separated by the self-cleavage of RiboJ. Decoy is designed to have a strong secondary structure so that it does not interact with other RNAs. Constitutive 3WJ trigger can repress Z with a strong inhibitory effect.

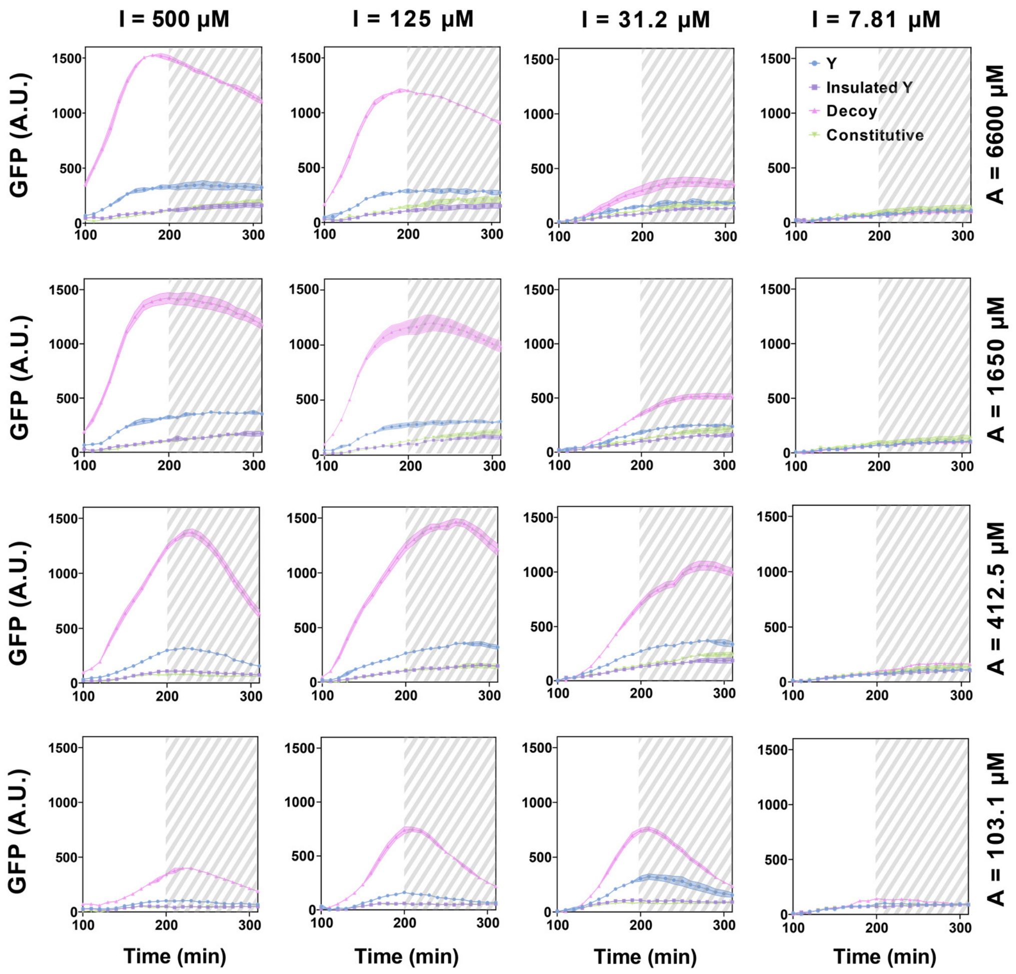

Supplementary Figure S3. *In vivo* characterization of the RNA-only IFFL circuit. Time course of GFP fluorescence measurements for different inducer concentrations; IPTG concentrations at 500 µM, 125 µM, 31.2 µM and 7.81 µM; Arabinose concentrations at 6600 µM, 1650 µM, 412.5 µM and 103.125 µM. Data for the first 90 mins are removed due to low OD600 values, and the time points beyond 200 mins are marked as gray dashed area to indicate the transition to stationary phase. Regular Y is represented by blue circles, insulated Y by purple squares, Decoy by pink triangles, and Constitutive by green triangles. The letters 'I' and 'A' represent IPTG and Arabinose, respectively. Relative errors for GFP fluorescence are from the standard deviation of three biological replicates.

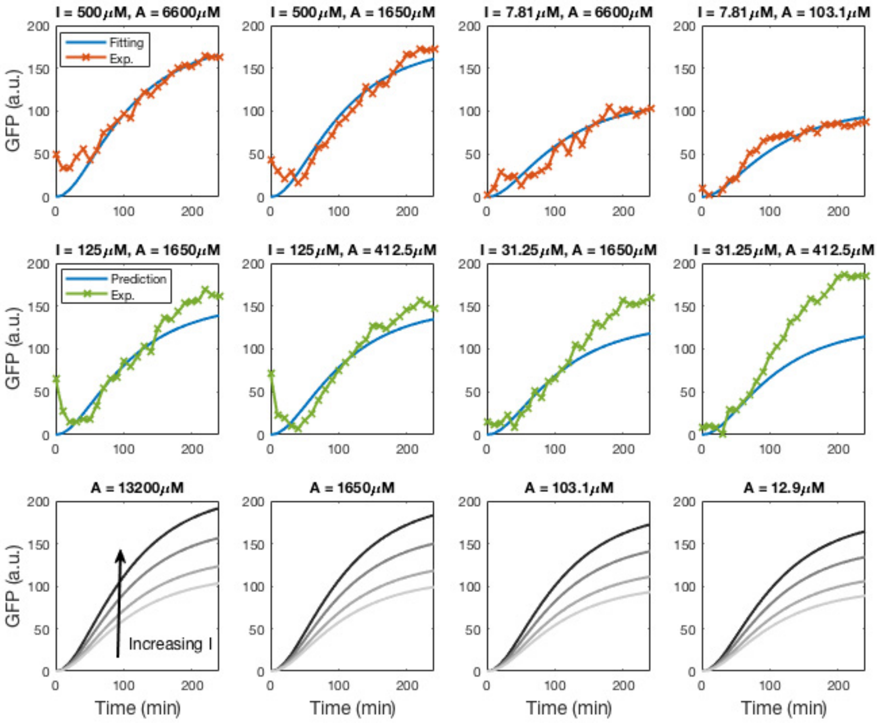

Supplementary Figure S4. RNA-only IFFL experiment and simulation. Top Row: experimental (solid red line with crosses) and model fitted (blue) GFP concentration. Middle Row: experimental (solid green line with crosses) and model predicted (blue) GFP concentration under new conditions. Bottom Row: model predicted GFP concentration for varying IPTG and Arabinose concentrations; the grayscale solid lines represent different IPTG concentrations at 2000 μM, 250 μM, 31.2 μM and 7.81 μM. The letters 'I' and 'A' represent IPTG and Arabinose, respectively.

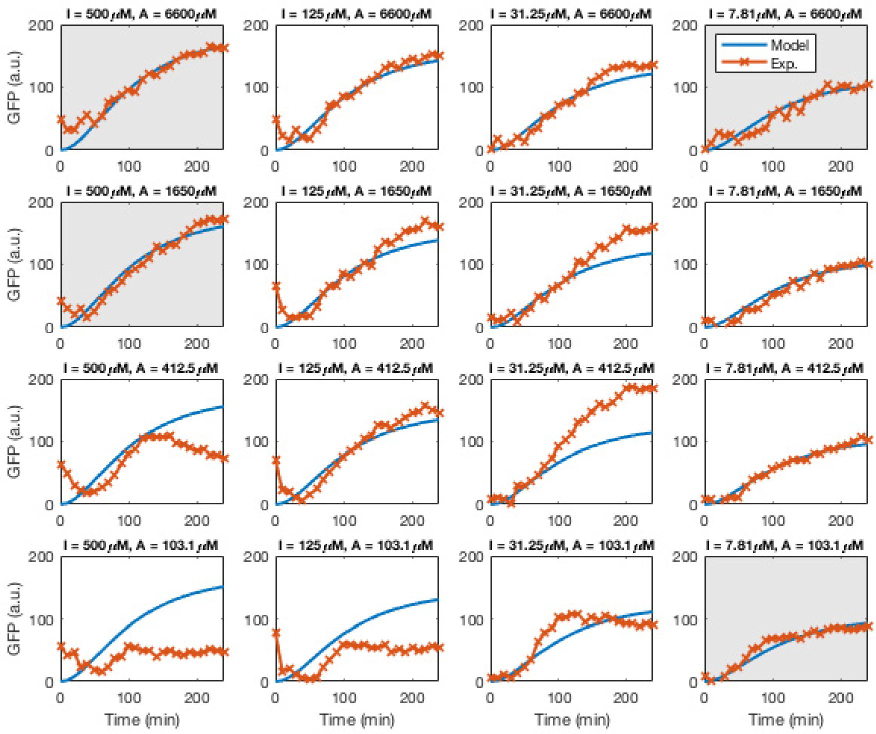

Supplementary Figure S5. RNA-only IFFL experiment and simulated GFP data for different IPTG and Arabinose concentrations. Solid red line with crosses: Experimental GFPs, Solid blue line: Simulated GFPs. Figure S4 shows the comparison between predicted and experimental GFP data for the RNA-only IFFL circuit for different IPTG and Arabinose concentrations. The four plots shaded in gray are the same one shown in the top row of Figure S4, where these experimental GFP data are used for estimating the model parameters. The remaining plots are the validation of the RNA-only model. In general, we can see that the model is able to capture the dynamics of the experimental GFP data with good predictive capability, indicating a good accuracy of the mechanistic model given by Equation (1) in the main text. The letters 'I' and 'A' represent IPTG and Arabinose, respectively.

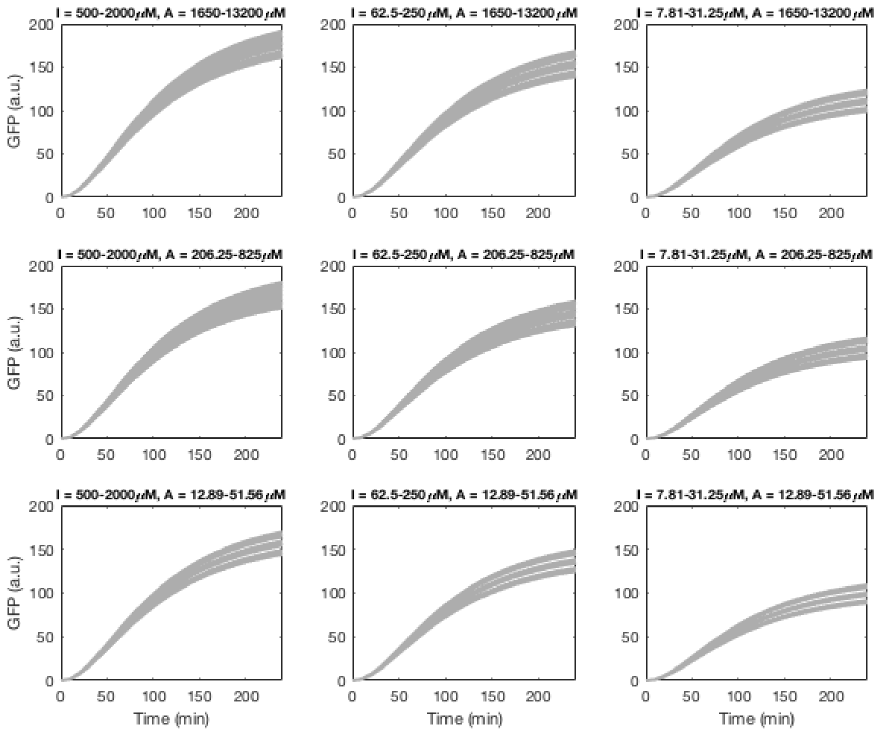

Supplementary Figure S6. Simulated RNA-only IFFL circuit for different combinations of IPTG and Arabinose concentrations. The purpose of this analysis is to determine whether any combinations of the inducers would generate a pulse. In this analysis, we also consider the IPTG and Arabinose concentrations that are outside the range considered in the experiment shown in Figure S3. From this analysis, we observe no pulse could be achieved with the RNA-only circuit, even if a wide range of inducer concentrations are considered. The letters 'I' and 'A' represent IPTG and Arabinose, respectively.

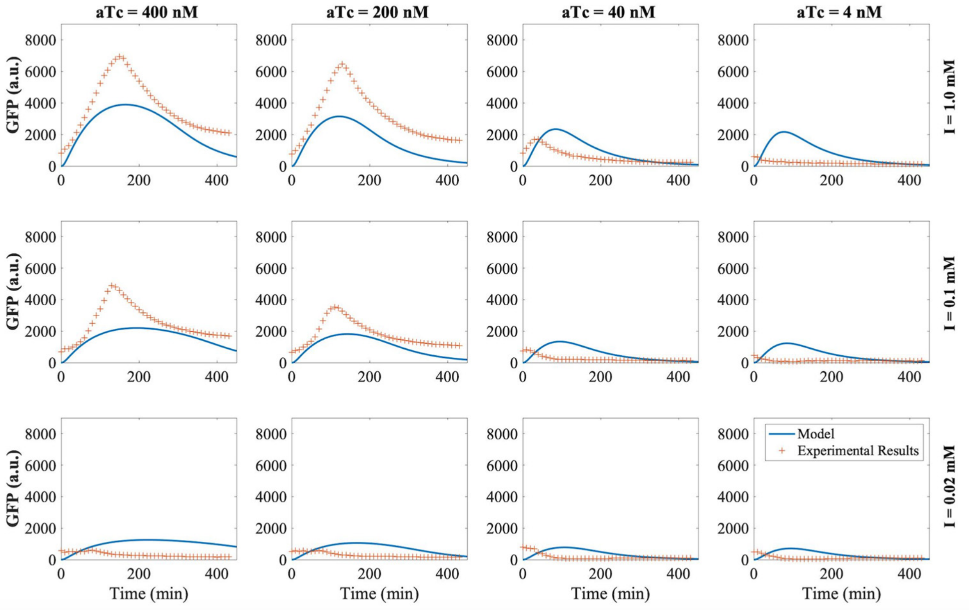

Supplementary Figure S7. RNA-protein hybrid IFFL simulation and experimental GFP data. The model was trained with 2 IPTG concentrations: 1mM and 0.1 mM. Including 2 aTc concentrations: 400 nM and 40 nM. The fitted model captures the pulse behavior observed in the experiments across the spectrum of aTc and IPTG values even though the training data set is relatively small. The letter 'I' represents IPTG.

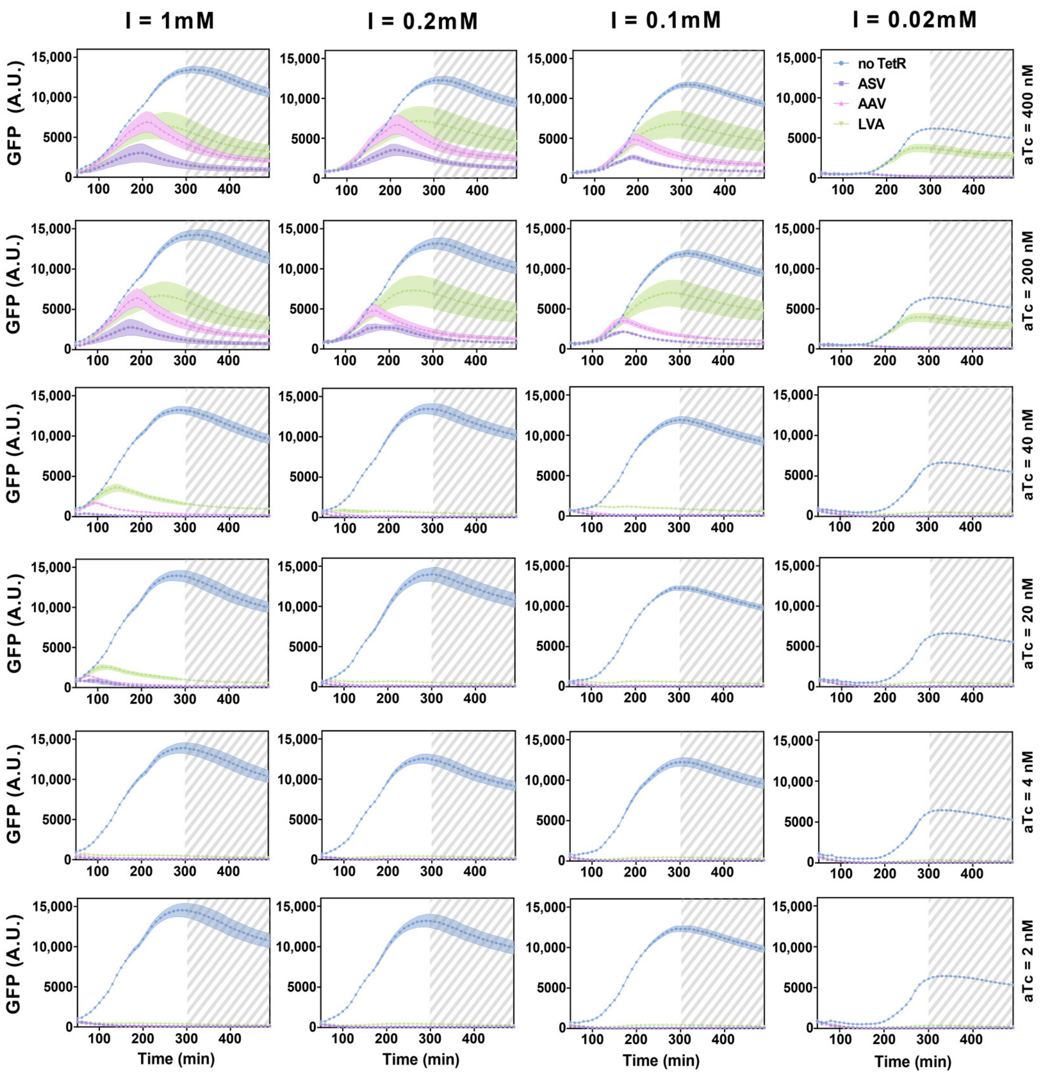

Supplementary Figure S8. Experimental results for RNA-protein hybrid circuit. Time course measurement of GFP fluorescence for different inducer concentrations; IPTG concentrations at 1 mM, 0.2 mM, 0.1 mM and 0.02 mM; aTc concentrations at 400 nM, 200 nM, 40 nM and 4 nM. Y is represented by different symbols; no TetR by blue circles, TetR-ASV by purple squares, TetR-AAV by pink triangles and TetR-LVA by green triangles. Data for the first 50 mins are removed due to low OD600 values, and the time points beyond 300 mins are marked as gray dashed area to indicate the transition to stationary phase. Relative errors for GFP fluorescence are from the s.d. of six biological replicates. The letter 'I' represents IPTG.

References

1. Chappell, J.; Takahashi, M.K.; Lucks, J.B. Creating small transcription activating RNAs. *Nat. Chem. Biol.* **2015**, *11*, 214-220, doi:10.1038/nchembio.1737.
2. Green, A.A.; Kim, J.; Ma, D.; Silver, P.A.; Collins, J.J.; Yin, P. Complex cellular logic computation using ribocomputing devices. *Nature* **2017**, *548*, 117–121.
3. Kim, J.; Zhou, Y.; Carlson, P.D.; Teichmann, M.; Chaudhary, S.; Simmel, F.C.; Silver, P.A.; Collins, J.J.; Lucks, J.B.; Yin, P. De novo-designed translation-repressing riboregulators for multi-input cellular logic. *Nat. Chem. Biol.* **2019**, *15*, 1173–1182.
4. Gibson, D.G.; Young, L.; Chuang, R.-Y.; Venter, J.C.; Hutchison, C.A.; Smith, H.O. Enzymatic assembly of DNA molecules up to several hundred kilobases. *Nat. Methods* **2009**, *6*, 343–345.
5. Andersen, J.B.; Sternberg, C.; Poulsen, L.K.; Bjørn, S.P.; Givskov, M.; Molin, S. New unstable variants of green fluorescent protein for studies of transient gene expression in bacteria. *Appl. Environ. Microbiol.* **1998**, *64*, 2240–2246.
6. Lagarias, J.C.; Reeds, J.A.; Wright, M.H.; Wright, P.E. Convergence Properties of the Nelder--Mead Simplex Method in Low Dimensions. *SIAM J. Optim.* **1998**, *9*, 112–147, doi:10.1137/s1052623496303470.
7. Bagh, S.; Mandal, M.; McMillen, D.R. Minimal genetic device with multiple tunable functions. *Phys. Rev. E* **2010**, *82*, 021911, doi:10.1103/PhysRevE.82.021911.

8. Zong, D.M.; Cinar, S.; Shis, D.L.; Josić, K.; Ott, W.; Bennett, M.R. Predicting Transcriptional Output of Synthetic Multi-input Promoters. *ACS Synth. Biol.* **2018**, *7*, 1834–1843, doi:10.1021/acssynbio.8b00165.
9. Ha, S.H.; Ferrell, J.E. Thresholds and ultrasensitivity from negative cooperativity. *Science* **2016**, *352*, 990–993, doi:10.1126/science.aad5937.
